# The epithelial-specific ER stress sensor ERN2/IRE1β enables host-microbiota crosstalk to affect colon goblet cell development

**DOI:** 10.1172/JCI153519

**Published:** 2022-09-01

**Authors:** Michael J. Grey, Heidi De Luca, Doyle V. Ward, Irini A.M. Kreulen, Katlynn Bugda Gwilt, Sage E. Foley, Jay R. Thiagarajah, Beth A. McCormick, Jerrold R. Turner, Wayne I. Lencer

**Affiliations:** 1 Division of Gastroenterology and Nutrition, Boston Children’s Hospital, Boston, Massachusetts, USA.; 2 Department of Pediatrics, Harvard Medical School, Boston, Massachusetts, USA.; 3 Harvard Digestive Disease Center, Boston Children’s Hospital, Boston, Massachusetts, USA.; 4 Department of Microbiology and Physiological Systems, and; 5 Program in Microbiome Dynamics, University of Massachusetts Chan Medical School, Worcester, Massachusetts, USA.; 6 Laboratory of Mucosal Barrier Pathobiology, Department of Pathology, Brigham and Women’s Hospital, Boston, Massachusetts, USA.; 7 Departments of Pathology and Medicine, Harvard Medical School, Boston, Massachusetts, USA.

**Keywords:** Gastroenterology, Cell stress, Inflammatory bowel disease

## Abstract

Epithelial cells lining mucosal surfaces of the gastrointestinal and respiratory tracts uniquely express ERN2/IRE1β, a paralogue of the most evolutionarily conserved endoplasmic reticulum stress sensor, ERN1/IRE1α. How ERN2 functions at the host-environment interface and why a second paralogue evolved remain incompletely understood. Using conventionally raised and germ-free *Ern2^–/–^* mice, we found that ERN2 was required for microbiota-induced goblet cell maturation and mucus barrier assembly in the colon. This occurred only after colonization of the alimentary tract with normal gut microflora, which induced *Ern2* expression. ERN2 acted by splicing *Xbp1* mRNA to expand ER function and prevent ER stress in goblet cells. Although ERN1 can also splice *Xbp1* mRNA, it did not act redundantly to ERN2 in this context. By regulating assembly of the colon mucus layer, ERN2 further shaped the composition of the gut microbiota. Mice lacking *Ern2* had a dysbiotic microbial community that failed to induce goblet cell development and increased susceptibility to colitis when transferred into germ-free WT mice. These results show that ERN2 evolved at mucosal surfaces to mediate crosstalk between gut microbes and the colonic epithelium required for normal homeostasis and host defense.

## Introduction

Epithelial cells lining mucosal surfaces of the gastrointestinal (GI) and respiratory tracts express 2 paralogues of the most evolutionarily conserved endoplasmic reticulum (ER) stress sensor IRE1. While all cell types express the essential stress sensor ERN1/IRE1α, which mediates one arm of the unfolded protein response to ER stress, only mucosal epithelial cells at these sites express ERN2/IRE1β (1). Why cells lining mucosal surfaces require a second IRE1 paralogue and how ERN2 contributes to the management of ER stress at the host-environment interface remain incompletely understood.

Several lines of evidence point to a role for ERN2 in goblet cells, which are specialized exocrine cells that secrete mucin glycoproteins and other host defense factors. In mice, expression of the *Ern2* gene is highly enriched in goblet cells of the small intestine (2), colon (3), and respiratory tract (4). Deletion of *Ern2* reduces the number of MUC2^+^ goblet cells in the ileum (5) and induces the accumulation of misfolded MUC2 precursors in secretory progenitor cells of the colon (6). These abnormal goblet cell phenotypes, however, are not present in mice with intestine-specific deletion of *Ern1*, implicating specificity for ERN2 function (5, 6). Mucin glycoproteins produced by goblet cells assemble into the extracellular mucus layers lining mucosal surfaces. These mucus layers provide the initial interface guarding the adjacent epithelium from environmental factors, including the high density of microbes colonizing the gut (7). Notably, in the colon, the colonizing gut microbes contribute to the regulation of the mucus barrier (8, 9) and the composition of the mucus barrier shapes the colonizing microbial communities by providing a niche for growth and attachment (10). How ERN2 contributes to the complex dynamic interface among microbes, the epithelium, and the mucus layers remains unknown.

ERN2, like ERN1, is an ER transmembrane protein with a lumenal stress-sensing domain and cytosolic kinase and endonuclease effector domains. The main signaling outputs for both proteins originate from the endonuclease domain, which enzymatically cleaves *Xbp1* mRNA in the cytosol to produce a spliced transcript coding for the transcription factor XBP1 (11, 12). The endonuclease domain can also degrade selected mRNAs to affect the cellular proteome through a process termed regulated IRE1-dependent decay of mRNA (or RIDD) (13, 14). Both endonuclease activities may play a role in how ERN2 regulates mucin biosynthesis (6, 15). But despite the high degree of sequence homology, ERN2 functions distinctly from ERN1 in that it has weaker endonuclease activity, responds only marginally to ER stress stimuli, and acts as a dominant-negative suppressor of stress-induced ERN1 signaling (16). We also note again that the functions of ERN2 cannot simply be redundant to ERN1. First, as noted above, the defects in goblet cell numbers and mucin biosynthesis found in *Ern2^–/–^* mice are not found in mice with intestine-specific deletion of *Ern1* (5, 6); second, the expression of *Ern2* but not *Ern1* gene expression is enriched in goblet cells lining mucosal surfaces; and third, other highly secretory cell types lack ERN2 but require ERN1 (e.g., pancreatic acinar and hematopoietic plasma cells).

Here, we used conventionally raised (CONV) (i.e., microbially colonized) and germ-free (GF) *Ern2^–/–^* mice to elucidate the function of ERN2 in the intestinal mucosa. We found that ERN2 enables goblet cell maturation, mucin secretion, and mucus barrier assembly. Remarkably, expression of ERN2 and function in the colon were dictated by the gut microbiota, and ERN2 in turn shaped the structure of the gut microbial communities. These results implicate ERN2 as a central node in mediating the host-microbiota crosstalk critical for development of the mucus barrier and host defense.

## Results

### ERN2 enables goblet cell development in response to the gut microbiota.

**Figure 1:**
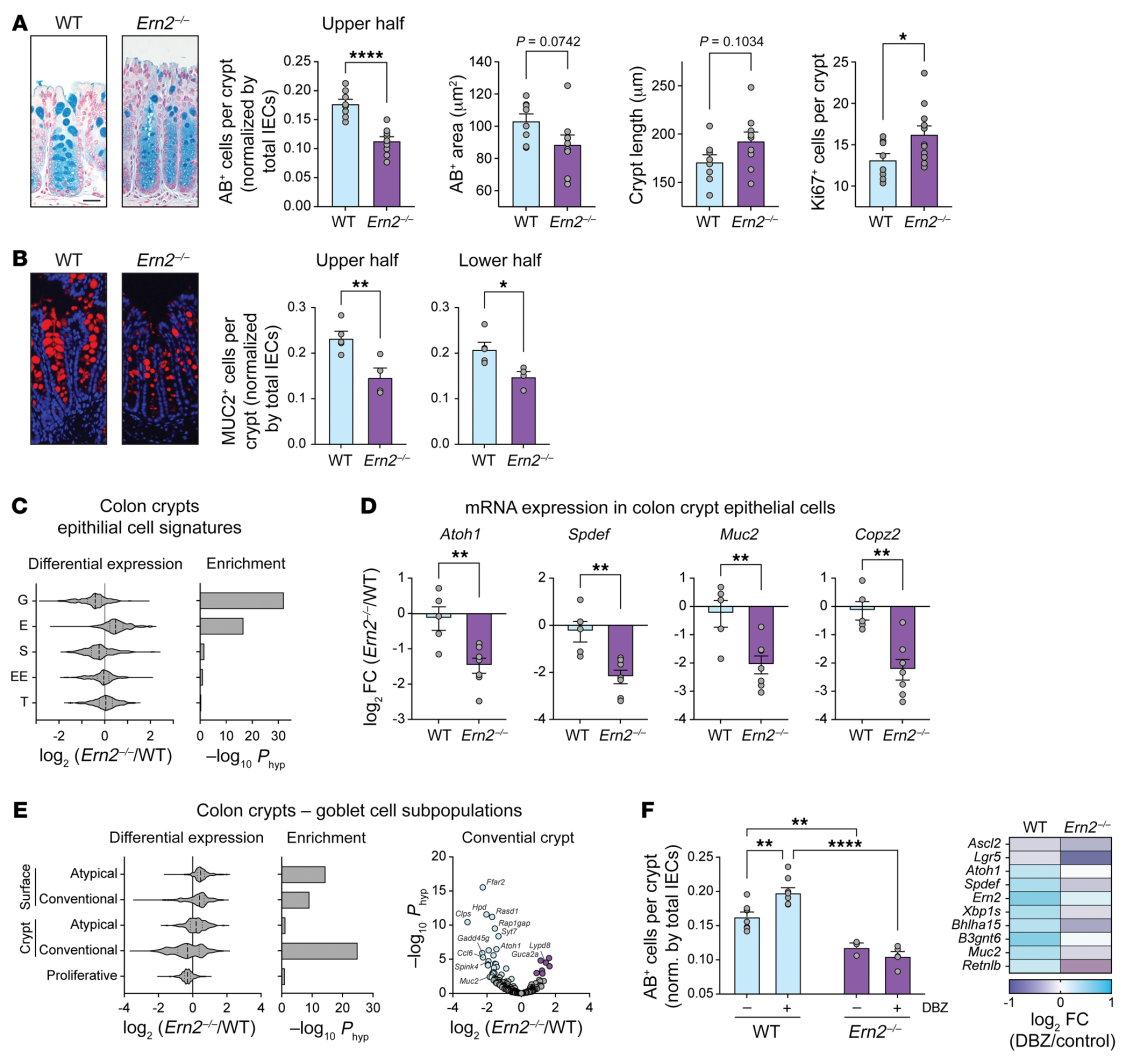
*Ern2^–/–^* mice have fewer goblet cells in distal colon under CONV conditions. (**A** and **B**) Representative images of (**A**) AB-stained and (**B**) anti-MUC2 antibody–stained sections of distal colon from CONV-WT and CONV-*Ern2^–/–^* mice. Bar graphs show AB^+^ cells and MUC2^+^ cells in the upper and lower half of crypts (normalized by number of crypt epithelial cells), AB-stained theca area, crypt length, and KI67^+^ cells per crypt in distal colon of WT and *Ern2^–/–^* littermates. Symbols represent average values for individual mice (WT, *n* = 8; *Ern2^–/–^*, *n* = 9) from 2 independent cohorts. Data are represented as mean ± SEM. Mean values were compared by unpaired *t* test. Scale bars: 50 μm. (**C**) Violin plot shows the distribution of relative mRNA expression of epithelial cell signature genes (2) in colon crypts. G, goblet; E, enterocyte; S, stem; EE, enteroendocrine; T, tuft. Bar graph shows enrichment of differentially expressed genes. (**D**) Bar graphs show relative mRNA expression in colon crypts measured by qPCR. Symbols represent individual mice, and data are represented by mean ± SEM. Mean values were compared by unpaired *t* test. (**E**) Left panels: Violin plot shows the distribution of relative mRNA expression for genes in goblet cell subpopulations (3), and bar graph shows enrichment of differentially expressed genes. Right panel: volcano plot shows differential expression of conventional crypt goblet cell genes. (**F**) Bar graph shows the number of AB^+^ cells in upper half of crypts (normalized by number of crypt epithelial cells) for mice treated with or without the γ-secretase inhibitor DBZ. Symbols represent the average values for individual mice, and data are represented as mean ± SEM. Mean values were compared by 2-way ANOVA (*n* = 4–8 mice per group). Heatmap shows differential mRNA expression in colon epithelial cells measured by qPCR. *P < 0.05; **P < 0.01; ****P < 0.0001.

To determine how ERN2 contributes to mucosal homeostasis, we first compared the tissue morphology and expression profiles of colon crypt epithelial cells from WT and *Ern2^–/–^* mice. The distal colon of CONV-*Ern2^–/–^* mice compared with CONV-WT controls had significantly fewer goblet cells with smaller mucus vacuoles, as assessed by Alcian blue (AB) (Figure 1A) and anti-MUC2 antibody staining (Figure 1B). Goblet cells in the upper half and lower half of crypts were equally affected by *Ern2* deletion (Figure 1B). In general, crypts were elongated with expanded proliferative (KI67^+^) zones in *Ern2^–/–^* mice, suggesting mild inflammation (Figure 1A), though not associated with apparent epithelial damage or immune cell infiltrate. Consistent with having fewer goblet cells, colon crypts from CONV-*Ern2^–/–^* mice had significant enrichment of differentially expressed goblet cell signature genes (Figure 1C, Supplemental Table 1, and Supplemental Data 1; supplemental material available online with this article; https://doi.org/10.1172/JCI153519DS1), including reduced mRNA expression for transcription factors, products, and genes related to secretory compartments that typify goblet cell function (Figure 1D). Of the recently described subpopulations of goblet cells in mouse distal colon (3), differential expression was most pronounced in conventional goblet cells residing in the crypts (Figure 1E). Similar histologic and molecular phenotypes were found in the proximal colon of *Ern2^–/–^* mice (Supplemental Figure 1, A–D). Unlike in CONV-WT mice, treatment of CONV-*Ern2^–/–^* mice with the γ-secretase inhibitor dibenzazepine (DBZ) to block Notch signaling and induce goblet cell differentiation (17) did not increase goblet cell numbers or upregulate goblet cell genes (Figure 1F). These data show that ERN2 is required for normal goblet cell development, likely by acting downstream of niche factors that normally signal for goblet cell differentiation in the colon.

**Figure 2:**
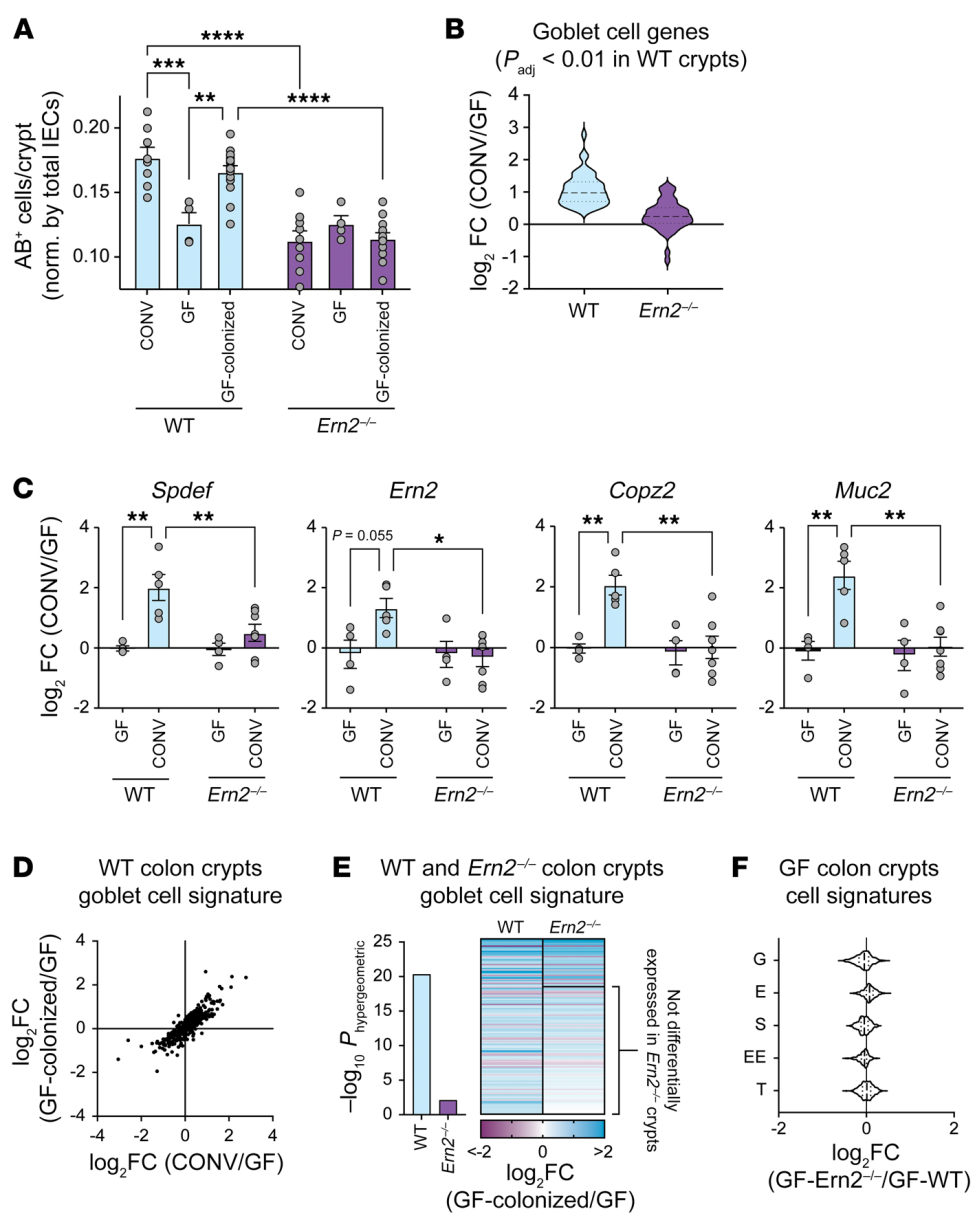
Gut microbes induce colon goblet cell development in an ERN2-dependent manner. (**A**) Bar graph shows the number of AB^+^ cells in the upper half of crypts in the distal colon of WT and *Ern2^–/–^* under CONV conditions, GF conditions, and GF followed by colonization with gut microbes from CONV-WT donor mice (Colonized). Symbols represent the average value for an individual animal, and data are represented as mean ± SEM. Mean values were compared by 2-way ANOVA. Data for CONV mice is from Figure 1A. (**B**) Violin plot showing relative mRNA expression for goblet cell signature genes upregulated in CONV-WT mice compared with GF-WT mice. Relative expression for those same genes is plotted for *Ern2^–/–^* mice. (**C**) Bar graphs show relative mRNA expression in colon crypts from WT and *Ern2^–/–^* mice for select genes measured by qPCR. Symbols represent individual mice, and data are represented as mean ± SEM. Mean values were compared by 2-way ANOVA. (**D**) Scatter plot compares differential expression of goblet cell signature genes for CONV-WT versus GF-WT compared with COLONIZED-WT versus GF-WT. (**E**) Bar graph shows enrichment of differentially expressed goblet cell signature genes for COLONIZED-WT and COLONIZED-*Ern2^–/–^* mice compared with GF controls. The heatmap shows the relative expression of differentially expressed genes. The subset of the genes not differentially expressed (*P_adj_* > 0.01) in *Ern2^–/–^* mice is indicated. (**F**) Violin plot showing the relative mRNA expression of epithelial cell signature genes from colon crypts of GF-*Ern2^–/–^* mice compared with GF-WT mice. *P < 0.05; **P < 0.01; ***P < 0.001; ****P < 0.0001.

Gut microbes affect goblet cell gene expression and numbers (18–21). To investigate whether this depends on ERN2, we rederived WT and *Ern2^–/–^* mice under GF conditions. CONV-WT mice had significantly more goblet cells per crypt compared with GF-WT mice (Figure 2A) as well as upregulation of *Ern2* and other goblet cell genes (Figure 2, B and C, Supplemental Table 2, and Supplemental Data 2). Also, as anticipated, colonization of GF-WT mice with a cecal microbiota from CONV-WT donor mice (COLONIZED-GF-WT) restored the number of goblet cells per crypt and differential expression of goblet cell genes to levels normally found in CONV-WT mice (Figure 2D, Supplemental Data 3, and Supplemental Table 3). This response was impaired in *Ern2^–/–^* mice. The presence of gut microbes in CONV-*Ern2^–/–^* mice or microbial colonized GF-*Ern2^–/–^* mice (COLONIZED-GF-*Ern2^–/–^*) had no detectable effect on the number of goblet cells (Figure 2A) or on goblet cell gene expression (Figure 2, B, C, and E, Supplemental Tables 4 and 5, and Supplemental Data 2 and 4). Consistent with these results, GF-WT and GF-*Ern2^–/–^* mice had nearly identical goblet cell numbers (Figure 2A) and crypt epithelial cell gene expression patterns (Figure 2F, Supplemental Table 6, and Supplemental Data 5). Thus, ERN2 is required for goblet cell development induced by gut microbes. This function of ERN2 is not fulfilled by ERN1 on its own, as *Ern2^–/–^* mice have normal *Ern1* expression levels. These results implicate a relationship between ERN2 and the gut microbiota in shaping the mucosal environment of the mouse colon.

### ERN2-mediated Xbp1 splicing and XBP1 are required to expand ER function and prevent ER stress in goblet cell maturation.

ERN2 could affect epithelial gene expression and goblet cell development by splicing *Xbp1* mRNA to produce the transcription factor XBP1. To test this, we analyzed gene expression profiles for colon crypt epithelial cells harvested from either WT or *Ern2^–/–^* mice. Colon crypts from both CONV-WT and COLONIZED-GF-WT mice had significant enrichment of upregulated genes associated with the transcription factor XBP1 compared with GF-WT mice (gProfiler TF:M01770_1 – CONV versus GF, –log_10_ adjusted *P* value [*P_adj_*] = 15.48; COLONIZED versus GF, –log_10_ *P_adj_* = 15.12). Microbial-induced enrichment of XBP1-associated genes, however, was not apparent in either CONV-*Ern2^–/–^* mice or GF-*Ern2^–/–^* mice following colonization with a cecal microbiota (COLONIZED-GF-*Ern2^–/–^*). Of the 132 goblet cell signature genes differentially expressed after microbial colonization of GF-WT mice, 96 were unchanged after colonization of GF-*Ern2^–/–^* mice (Figure 2E and Supplemental Data 4). These genes were significantly associated with XBP1, ER function, protein processing in the ER, and ER-to-Golgi transport (Table 1). These results implicate ERN2 and its downstream effector XBP1 in mediating microbial-induced gene expression and goblet cell maturation in the colon.

**Figure 3:**
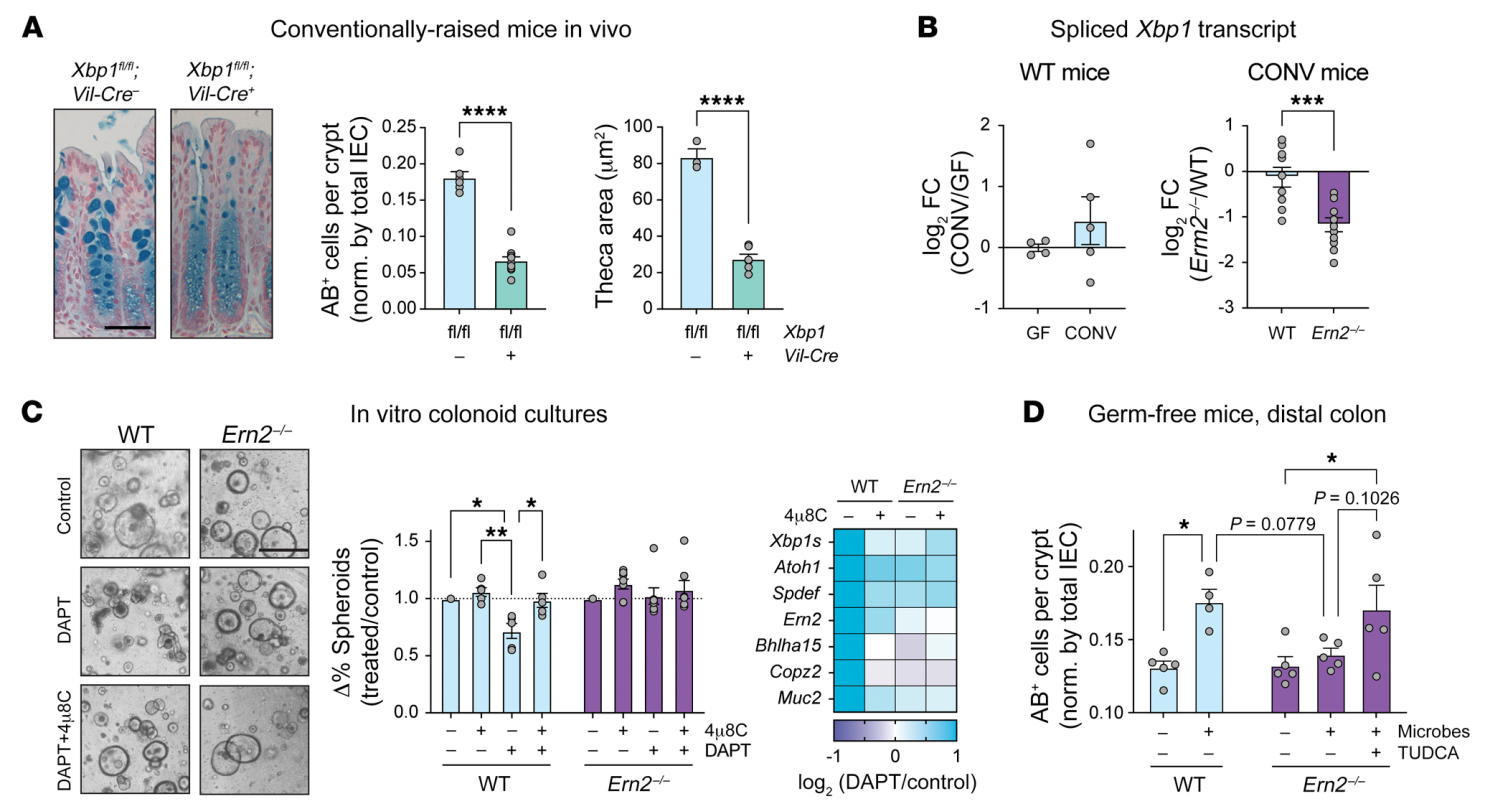
ERN2-mediated *Xbp1* splicing and XBP1 expand ER function and prevent ER stress for goblet cell maturation. (**A**) Representative images of AB-stained sections of distal colon from *Xbp1^fl/fl^*;*Vil-Cre*^–^ (*n* = 6) and *Xbp1^fl/fl^*;*Vil-Cre*^+^ (*n* = 12) littermates. Bar graphs show the number of AB^+^ cells in the upper half of crypts and the AB-stained goblet cell theca area. Symbols represent individual mice, and data are represented as mean ± SEM. Mean values were compared by unpaired *t* test. Scale bar: 50 μm. (**B**) Bar graph shows relative expression of spliced *Xbp1* transcript in colon crypts from (left panel) GF-WT (*n* = 4) and CONV-WT mice (*n* = 6) and (right panel) CONV-WT (*n* = 9) and CONV-*Ern2^–/–^* (*n* = 11) mice. Symbols represent individual mice, and data are represented as mean ± SEM. Mean values were compared by unpaired *t* test. (**C**) Left panel: representative images of mouse colonoids treated with the γ-secretase inhibitor DAPT in the presence or absence of the IRE1 inhibitor 4μ8C. Scale bar: 500 μm. Center panel: differentiation status was assayed by scoring spheroid versus nonspheroid morphology. Bar graph shows the change in the percentage of colonoids with spheroid morphology relative to untreated controls for a given colonoid line within an experiment. Symbols represent independent experiments (WT, *n* = 5; *Ern2^–/–^*, *n* = 7). Mean values within genotypes were compared by 2-way ANOVA. Right panel: heatmap shows relative mRNA expression for select genes measured by qPCR from a single experiment. (**D**) Bar graph shows the number of AB^+^ cells in upper half of well-defined crypts following colonization of GF-*Ern2^–/–^* mice with a microbiota from CONV-WT donor mice in the presence or absence of TUDCA. Symbols represent the average value for an individual mouse, and data are represented as mean ± SEM (WT: GF/COLONIZED, *n* = 5/4; *Ern2^–/–^*: GF/COLONIZED/COLONIZED+TUDCA, *n* = 5/5/5). Mean values were compared by 1-way ANOVA. *P < 0.05; **P < 0.01; ***P < 0.001; ****P < 0.0001.

To determine whether XBP1 contributes to goblet cell development, we analyzed the colonic epithelium of CONV mice with intestine-specific deletion of *Xbp1* (*Xbp1^fl/fl^*;*Vil-Cre*^+^). CONV-*Xbp1^fl/fl^*;*Vil-Cre*^+^ mice phenocopied CONV-*Ern2^–/–^* mice in that they had fewer AB^+^ cells per crypt (Figure 3A) and reduced expression of goblet cell signature genes compared with *Xbp1^fl/fl^*;*Vil-Cre*^–^ controls (Supplemental Figure 2B). Thus, XBP1 is required for goblet cell development in the colon. To determine whether XBP1 acts as a downstream effector of ERN2 in vivo, we measured spliced *Xbp1* mRNA, which is needed for XBP1 translation (11, 12). Spliced *Xbp1* mRNA, which was stimulated by gut microbes in WT mice, was significantly reduced in CONV-*Ern2^–/–^* mice (Figure 3B). Expression of Flag-tagged ERN2 in HEK293 cells induced *Xbp1* splicing (16) and XBP1-dependent gene expression — including that of genes associated with ER function, protein folding, and ER-to-Golgi vesicle trafficking that underlie the secretory cell phenotype (Supplemental Figure 2C, Supplemental Data 6, and Supplemental Tables 8 and 9). Expression of these genes was decreased in colon crypt epithelial cells of CONV-*Ern2^–/–^* mice (Supplemental Figure 2D). To determine whether ERN2 *Xbp1* splicing is required for goblet cell differentiation, we used a colonoid differentiation assay. Treatment of WT colonoids with the γ-secretase inhibitor DAPT decreased the percentage of colonoids with a spheroid (stem cell) morphology and increased expression of differentiation-dependent goblet cell marker genes (Figure 3C). *Ern2^–/–^* colonoids, on the other hand, did not lose spheroid morphology following DAPT treatment. Furthermore, the endonuclease activity of ERN2 was required, as the IRE1 inhibitor 4μ8C (22) blocked DAPT-induced goblet cell differentiation in WT colonoids (Figure 3C). Thus, goblet cell development in the mouse colon depends upon expression of ERN2, its function in enzymatically splicing *Xbp1* mRNA, and XBP1 as downstream effector.

As XBP1 normally expands ER function to enable proteostasis, depletion of XBP1 in *Ern2^–/–^* mice may result in ER stress following colonization. Crypts from COLONIZED-GF-*Ern2^–/–^* mice had significant enrichment of differentially expressed genes associated with the response to ER stress (Gene Ontology [GO]:0034976, –log_10_ *P_adj_* = 5.15), with *Derl3*, *Chac1*, and *Trib3* being the most upregulated genes in crypts of COLONIZED-GF-*Ern2^–/–^* mice compared with COLONIZED-GF-WT mice (Supplemental Table 7 and Supplemental Data 7). COLONIZED-GF-*Ern2^–/–^* mice also had decreased expression of genes involved in protein translation (GO:0006412, –log_10_ *P_adj_* = 18.23) and ribosome function (GO:0003735, –log_10_ *P_adj_* = 34.79), suggesting that *Ern2^–/–^* crypt epithelial cells have slowed protein synthesis in response to ER stress following colonization. Similarly, colon epithelial cells from CONV-*Ern2^–/–^* mice had elevated levels of ER chaperones and ER-associated degradation (ERAD) machinery consistent with ER stress (Supplemental Figure 1). Treatment of mice with antibiotics to deplete gut microbes significantly attenuated ER stress markers in CONV-*Ern2^–/–^* mice (Supplemental Figure 2D). To determine whether resolution of ER stress can enable normal goblet cell development in response to microbes, we colonized GF-*Ern2^–/–^* in the presence of the chemical chaperone tauroursodeoxycholic acid (TUDCA) (23, 24). TUDCA treatment enabled the induction of AB^+^ goblet cells following colonization to an extent similar to that of colonization of GF-WT mice (Figure 3D). From these results, we conclude that ERN2 is required for the maturation of goblet cells in response to gut microbes, acting by expanding ER and secretory compartment function to prevent the accumulation of ER stress.

### Ern2^–/–^ mice have impaired mucus barrier assembly and host defense.

**Figure 4:**
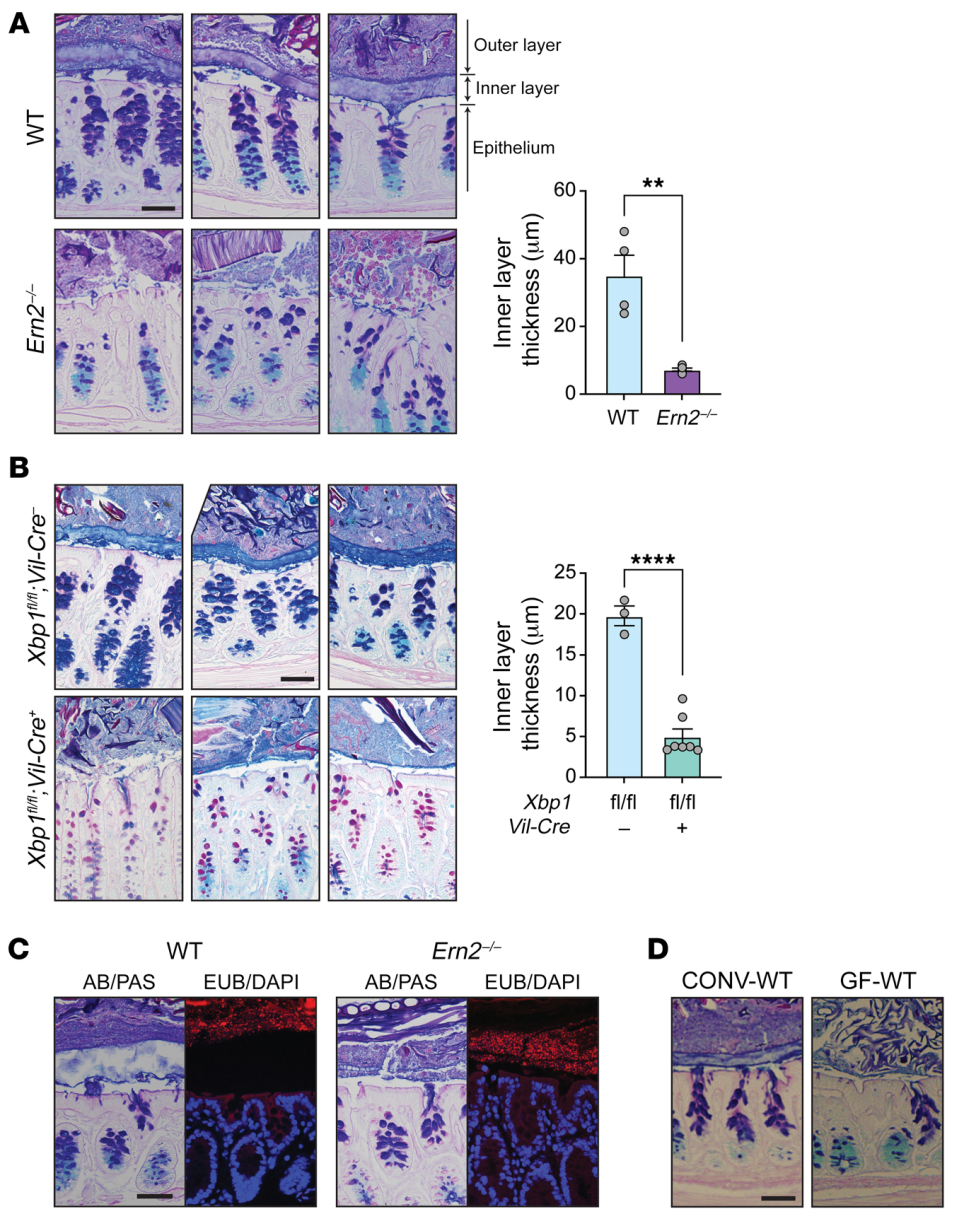
Impaired assembly of the colon mucus layer in *Ern2^–/–^* mice. Representative images of the colon mucus layer assessed by AB/PAS staining of Carnoy’s fixed tissue from (**A**) CONV-WT and CONV-*Ern2^–/–^* mice and (**B**) *Xbp1^fl/fl^*;*Vil-Cre*^–^ and *Xbp1^fl/fl^*;*Vil-Cre*^+^ mice. Bar graphs show the thickness of the inner mucus layer. Symbols represent the average value of the distribution of measures around at least 2 full cross sections for individual mice (WT, *n* = 4; *Ern2^–/–^*, *n* = 5; *Xbp1^fl/fl^*;*Vil-Cre*^–^, *n* = 3; *Xbp1^fl/fl^*;*Vil-Cre*^+^, *n* = 7). Data are represented as mean ± SEM. Mean values were compared by unpaired *t* test. **P < 0.01; ****P < 0.0001. (**C**) Representative images of Carnoy’s fixed colon tissue from CONV-WT and CONV-*Ern2^–/–^* mice stained with AB/PAS or the 16S rRNA in situ hybridization probe EUB338 (red) to detect lumenal bacteria. Nuclei were stained with DAPI (blue). (**D**) Representative images of Carnoy’s fixed tissue from CONV-WT and GF-WT mice stained with AB/PAS. Scale bars: 50 μm.

Goblet cells secrete mucin glycoproteins that assemble into mucus layers protecting the epithelium. In the distal colon, this includes a dense inner mucus layer that is impenetrable to microbes (25) and a second loosely attached outer mucus layer that is colonized by microbes. To determine whether ERN2 affects mucus assembly, we analyzed the mucus layers in Carnoy’s fixed colonic sections from CONV-WT and CONV-*Ern2^–/–^* mice. Consistent with a defect in goblet cell development, the distal colon of CONV-*Ern2^–/–^* mice had impaired mucus assembly with a significantly thinner inner mucus layer compared with that of CONV-WT mice (Figure 4A). As a result, gut microbes were abnormally located immediately adjacent to the surface epithelial cells (Figure 4C). Similar defects were found in mice with intestine-specific deletion of *Xbp1* (Figure 4B). As reported previously (8, 21, 26), formation of the inner mucus layer was microbe dependent (Figure 4D). This further links ERN2 with microbiota-induced goblet cell development.

**Figure 5:**
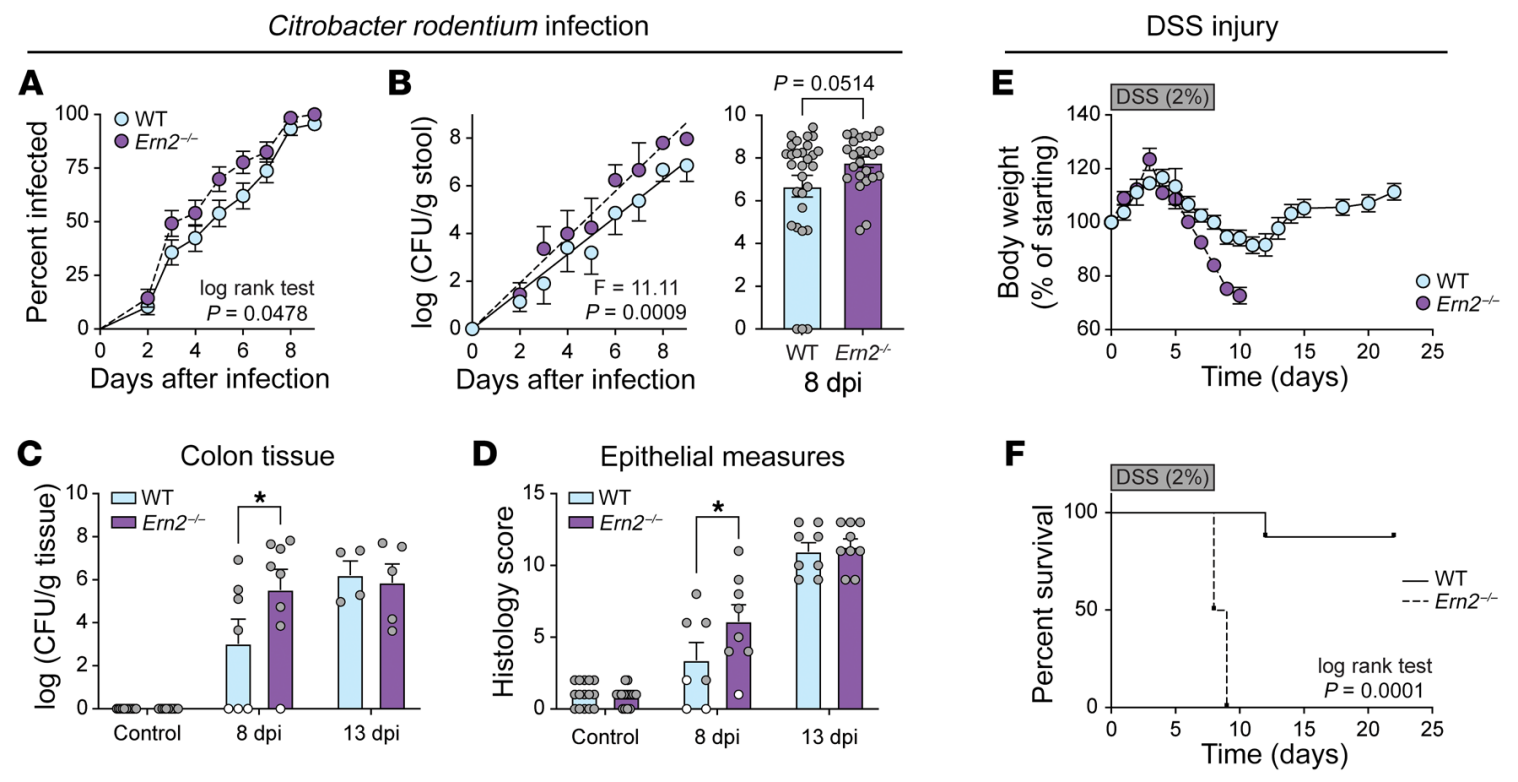
*Ern2^–/–^* mice have increased susceptibility to *C. rodentium*–and DSS-associated colitis. (**A** and **B**) Time courses represent the onset of infection monitored by (**A**) the percentage of mice with *C*. *rodentium* detected in stool and (**B**) *C*. *rodentium* CFUs in stool. Time courses represent the average measured in 47 WT and 38 *Ern2^–/–^* mice from 5 independent experiments. (**B**) Bar graph shows *C*. *rodentium* CFUs in stool of infected mice at 8 days after infection. Symbols represent individual mice. Data are represented as mean ± SEM. Mean values were compared by unpaired *t* test. (**C** and **D**) Bar graphs show *C*. *rodentium* CFUs cultured from colon tissue and (**D**) histology scores of epithelial damage in control mice and infected mice at 8 and 13 days after infection. Symbols represent individual mice. Data are represented as mean ± SEM. Mean values were compared by 2-way ANOVA. (**E** and **F**) Time courses shows (**E**) change in body weight and (**F**) survival for WT and *Ern2^–/–^* mice during administration of DSS for 8 days followed by recovery for 14 days. Symbols represent average values (WT, *n* = 8; *Ern2^–/–^*, *n* = 8). Survival curves were compared using a log rank test. *P < 0.05.

We tested to determine whether impaired mucus assembly in *Ern2^–/–^* mice affected host defense to the mouse pathogen *Citrobacter rodentium* and the detergent dextran sulfate sodium (DSS) — both models depend on the colonic mucus barrier (26–28). CONV-*Ern2^–/–^* mice had an earlier onset of *C*. *rodentium* infection (Figure 5, A and B). At 8 days after infection, *Ern2^–/–^* mice had approximately 10-fold higher levels of pathogen in stool (Figure 5B). This was associated with more adherent *C*. *rodentium* cultured from colon tissue (Figure 5C) and a greater extent of epithelial damage in CONV-*Ern2^–/–^* mice compared with CONV-WT mice (Figure 5D). After peak levels of infection were attained (e.g., 13 days after infection), WT and *Ern2^–/–^* mice had similar degrees of tissue colonization and epithelial damage (Figure 5, C and D). Consistent with previous studies (1), *Ern2^–/–^* mice were also more susceptible to DSS-associated colitis. *Ern2^–/–^* mice treated with DSS had earlier onset and more pronounced weight loss (Figure 5E) and significantly impaired survival (Figure 5F) compared with WT mice. Thus, ERN2 is required for proper assembly of the mucus barrier that protects the epithelium from infection and chemical injury.

### ERN2 mediates a microbiota-epithelial-mucus feedback loop that maintains mucosal homeostasis.

**Figure 6:**
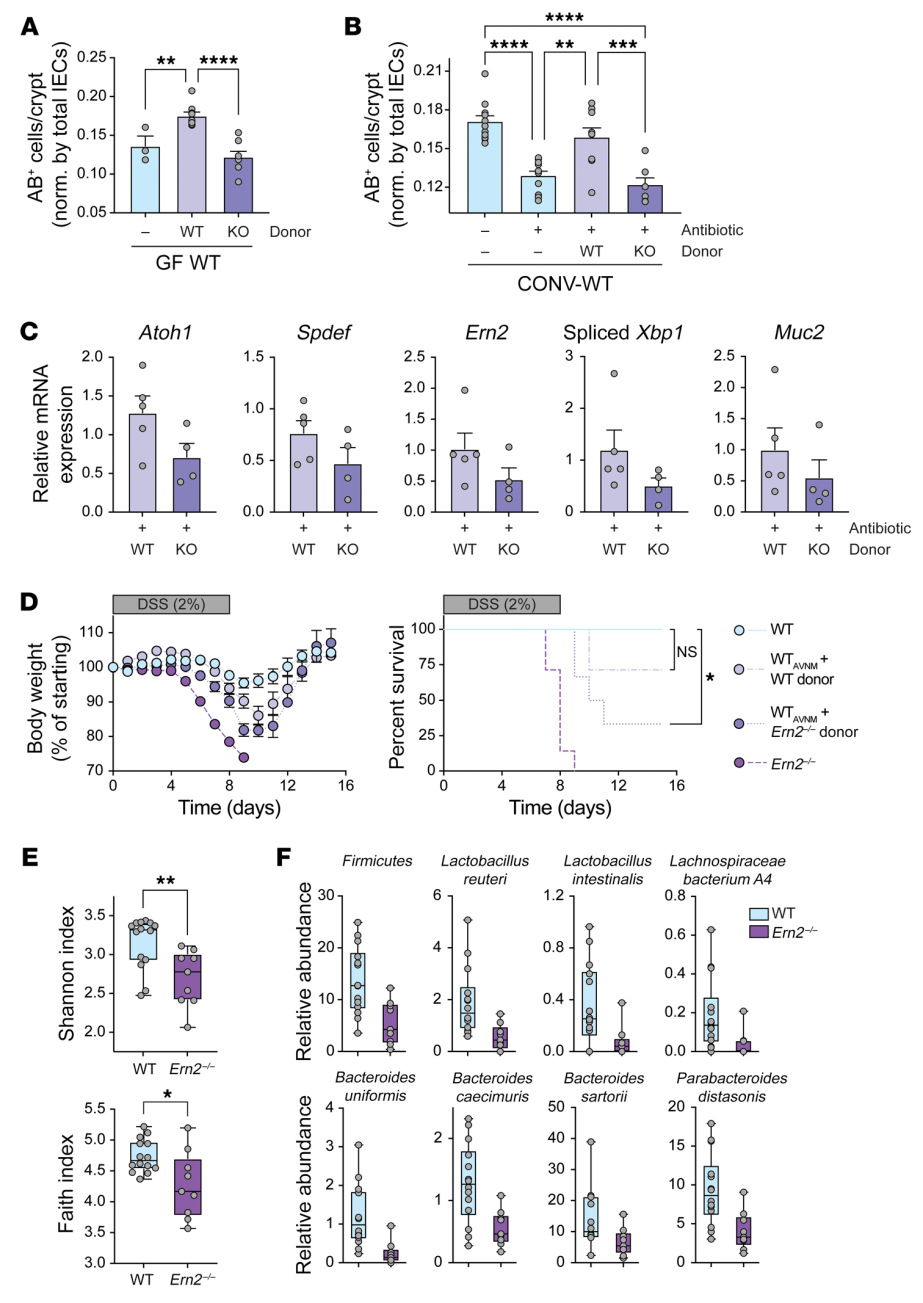
*Ern2^–/–^* microbiota are unable to support goblet cell development when transferred into WT recipient mice. Bar graphs show number of AB^+^ cells per crypt in the upper half of crypts in the distal colon of (**A**) GF-WT mice (*n* = 3) and GF-WT mice colonized with microbiota from WT (*n* = 9) or *Ern2^–/–^* (*n* = 8) donor mice and (**B**) CONV-WT mice (*n* = 11), antibiotic-treated CONV-WT mice (*n* = 11), and antibiotic-treated CONV-WT mice that were cohoused with WT (*n* = 9) or *Ern2^–/–^* (*n* = 7) donor mice. Symbols represent individual mice. Data are represented as mean ± SEM. Mean values were compared by 1-way ANOVA. (**C**) Bar graphs show relative mRNA expression for indicated genes measured by qPCR in colon epithelial cells from antibiotic-treated WT mice that were cohoused with either WT (*n* = 5) or *Ern2^–/–^* (*n* = 4) donor mice. Expression levels are shown relative to control WT mice (no antibiotics, no cohousing). (**D**) Time courses show (left panel) change in body weight and (right panel) survival during administration of DSS for 8 days followed by 14 days of recovery for WT (*n* = 6), *Ern2^–/–^* (*n* = 7), and antibiotic-treated WT mice cohoused with WT (*n* = 7) or *Ern2^–/–^* donors (*n* = 6). Survival curves were compared using log rank test. (**E**) Box plots show α diversity indices for microbiota from WT and *Ern2^–/–^* mice. Symbols represent values for individual mice, and error bars represent minimum and maximum. (**F**) Box plots show relative abundance data for indicated taxa that are significantly different between microbiota from CONV-WT and CONV-*Ern2^–/–^* mice. Symbols represent relative abundance for an individual mouse. FDR *q* values were calculated with MaAsLin2 (63) (WT, *n* = 14; *Ern2^–/–^*, *n* = 9). *P < 0.05; **P < 0.01; ***P < 0.001; ****P < 0.0001.

Gut microbes and the colonic mucus layer are linked via a feedback loop, where the microbiota induce mucus barrier formation and the mucus layer and composition provide a niche for colonizing microbes. We hypothesized that the microbiota colonizing WT and *Ern2^–/–^* mice might be different and may differentially contribute to the *Ern2^–/–^* goblet cell phenotype. To test this, we first asked whether the microbiota from *Ern2^–/–^* mice could phenocopy the microbiota from WT mice by inducing goblet cell development when transferred into recipient GF-WT mice (as in Figure 2A). We also tested this using CONV-WT recipient mice pretreated with antibiotics to deplete the gut microbiota. In both models, we found that colonization of GF-WT or antibiotic-treated CONV-WT mice with microbiota from CONV-WT donor mice fully restored goblet cell numbers as expected (Figure 6, A and B). In contrast, the microbiota from CONV-*Ern2^–/–^* donor mice failed to rescue the normal goblet cell phenotype in WT recipients (Figure 6, A and B). Notably, recipients of the *Ern2^–/–^* microbiota had decreased expression of goblet cell marker genes (including *Ern2*, Figure 6C) and increased susceptibility to DSS-associated colitis (Figure 6D).

To determine the impact of ERN2 on the microbiota, we used metagenomic sequencing to compare the composition of microbes in stool obtained from CONV-WT and CONV-*Ern2^–/–^* mice. The microbiota from *Ern2^–/–^* mice was less diverse than that of WT mice (Figure 6E), and the taxa of the 2 microbial communities were significantly different (weighted UniFrac, *P* = 0.012; Bray-Curtis dissimilarity, *P* = 0.005; Jaccard distance, *P* = 0.007). The *Ern2^–/–^* microbiota had significant reduction in the relative abundance of taxa belonging to the *Firmicutes* phylum overall as well as species known to promote mucus barrier function (e.g., *Lactobacillus reuteri*; ref. 29, Figure 6F, and Supplemental Data 8). Thus, loss of ERN2 function results in a dysbiotic gut microbiota that is unable to promote *Ern2* expression and goblet cell development when transferred into WT recipient mice.

**Figure 7:**
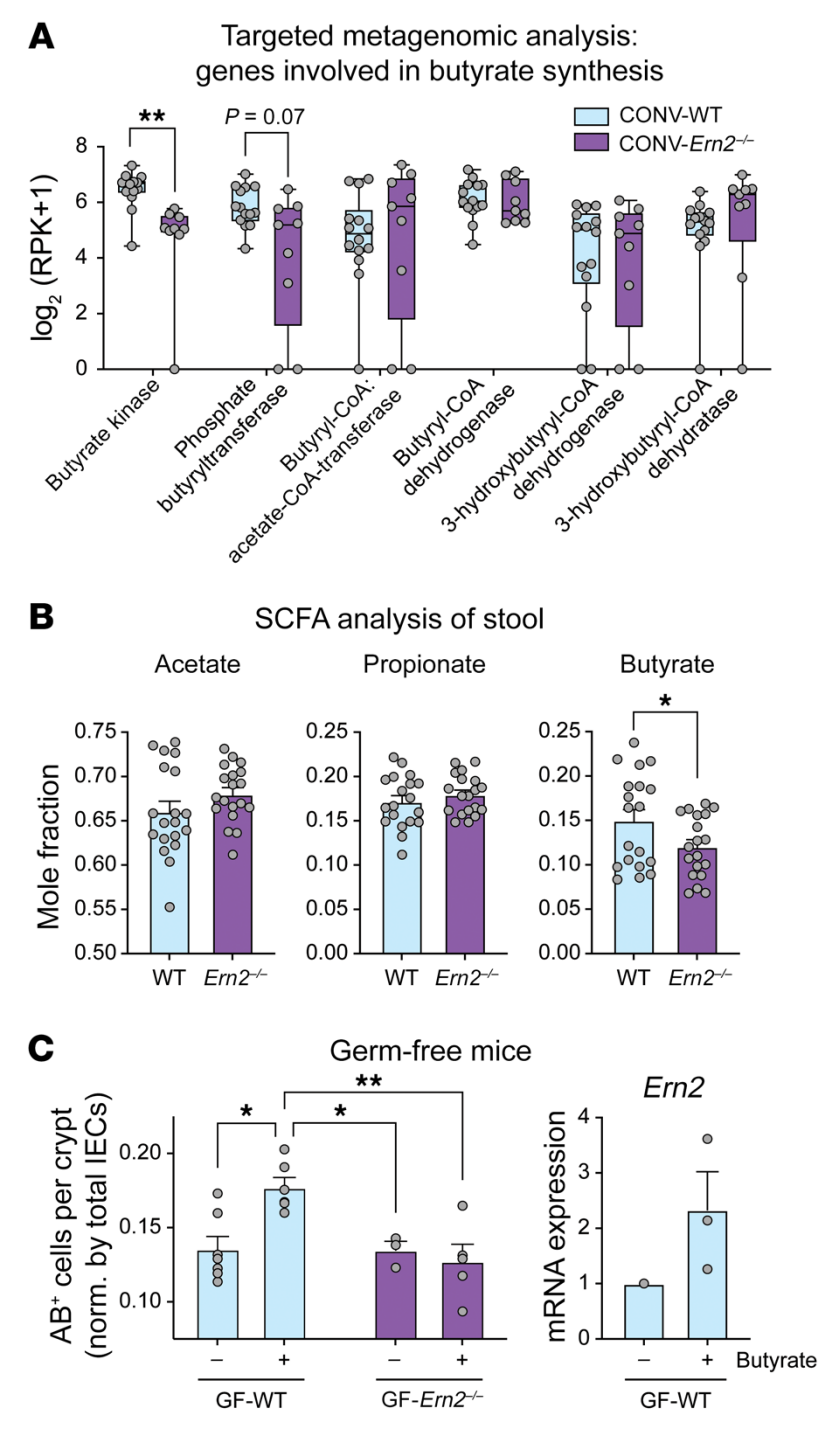
The SCFA butyrate links gut microbes with *Ern2* expression. (**A**) Box plots show whole genome sequence reads for enzymes in butyrate metabolism measured in stool from WT (*n* = 14) and *Ern2^–/–^* (*n* = 9) mice. Symbols represent an individual mouse, and bars represent the range. Mean values were compared using multiple *t* tests. (**B**) Bar graphs show mole fraction of SCFAs measured in stool from WT (*n* = 19) and *Ern2^–/–^* (*n* = 19) mice. Values are shown as the mole fraction of each relative to the total SCFAs measured in each sample. Symbols represent an individual mouse. Data are represented as mean ± SEM. (**C**) Left panel: bar graph shows the number of AB^+^ cells in the upper half of crypts in the distal colon of GF-WT and GF-*Ern2^–/–^* mice that received regular drinking water or drinking water supplemented with sodium butyrate. Symbols represent the average value for an individual mouse. Data are represented as mean ± SEM. Mean values were compared by 2-way ANOVA. Right panel: bar graph shows relative expression of *Ern2* mRNA measured by qPCR in colon crypt epithelial cells from GF-WT mice (control, *n* = 1; butyrate *n* = 3). *P < 0.05; **P < 0.01.

To understand what components of the gut microbiota modulate *Ern2* expression and goblet cell development, we analyzed the metagenomes for metabolic functions depleted in *Ern2^–/–^* mice. Given the reduced representation of *Firmicutes*, we initially asked whether butyrate metabolism was altered in the *Ern2^–/–^* microbiota. In a targeted analysis, the metagenome of CONV-*Ern2^–/–^* mice had reduced representation of butyrate kinase and phosphate butyryltransferase genes (Figure 7A) — enzymes required for butyrate production. This was associated with reduction in the mole fraction of butyrate detected in stool from *Ern2^–/–^* mice (Figure 7B). Treatment of polarized T84 cell monolayers or LS174T cells with 10 or 1 mM butyrate, respectively, increased *Ern2* mRNA expression as well as spliced *Xbp1* mRNA (Supplemental Figure 3). Since these concentrations were lower than in the mouse colon (30–32), we also tested butyrate in vivo. GF-WT mice given drinking water with butyrate (100 mM) (33) had an increase in number of goblet cells to levels induced by colonization with a microbiota from CONV-WT donor mice, with a corresponding increase in *Ern2* mRNA in colon crypt epithelial cells. In contrast, there was no effect on goblet cell numbers in GF-*Ern2^–/–^* mice (Figure 7C). Thus, we conclude that butyrate is a component of the gut microbiota that regulates *Ern2* expression and goblet cell development in the colon.

### ERN2 association with ulcerative colitis and goblet cell development in primary human colonoids.

**Figure 8:**
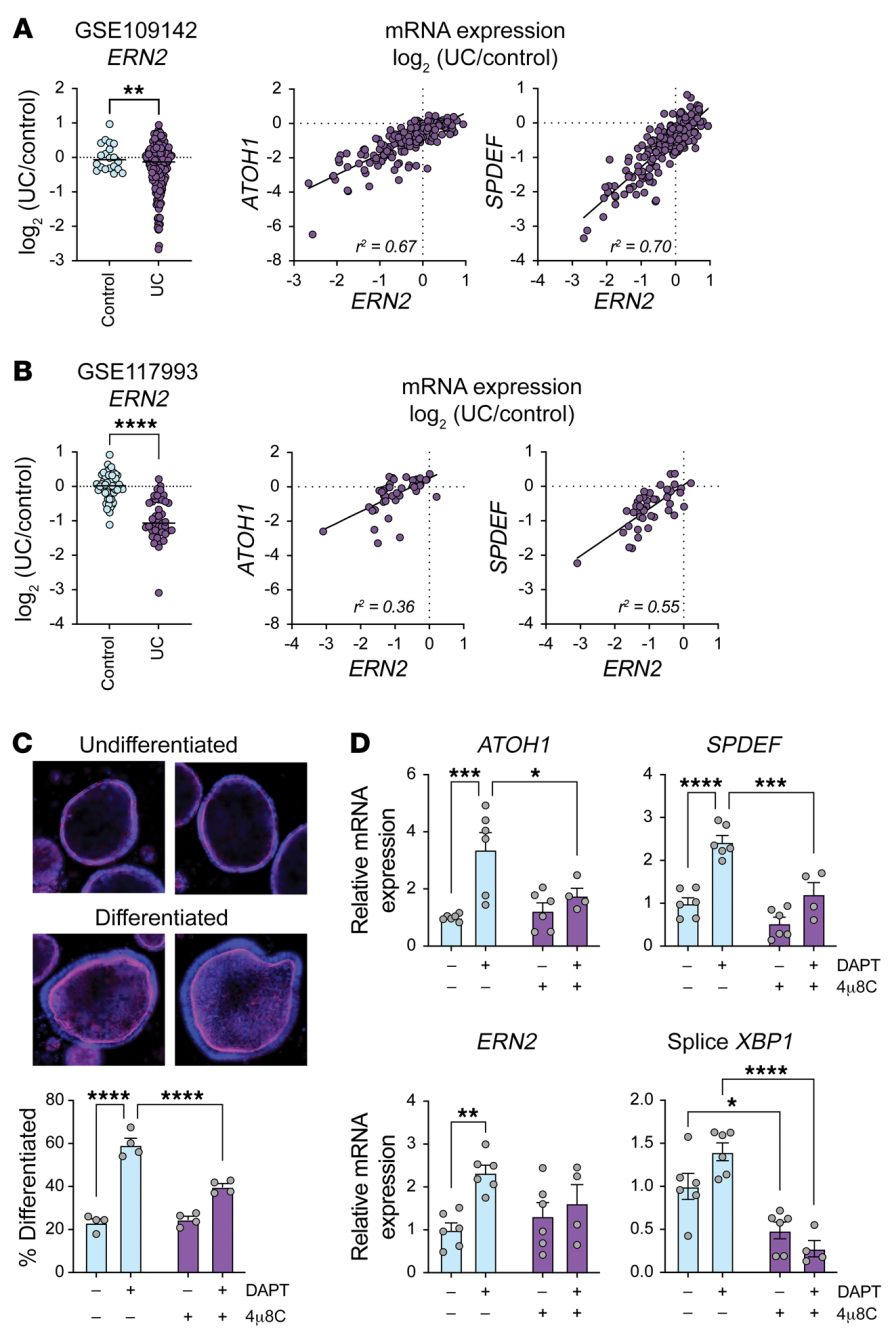
ERN2 expression is associated with UC and goblet cell development in primary human colonoids. (**A** and **B**) Left panels: scatter plots show relative mRNA expression for *ERN2* in rectal biopsies from individuals with and without UC. Right panels: scatter plots show correlation between mRNA expression levels of ERN2 and goblet cell transcription factors ATOH1 or SPDEF in individuals with UC. (**C**) Representative images of undifferentiated and differentiated human colonoids. Fixed colonoids were stained with DAPI (blue) and phalloidin (magenta). Differentiated and undifferentiated colonoids were quantitated in cultures treated with DAPT and the IRE1 inhibitor 4μ8C. Symbols represent individual biological replicates from 3 independent experiments. Data are represented as mean ± SEM. Mean values were compared by 2-way ANOVA. Original magnification, ×10. (**D**) Bar graphs show relative mRNA expression for *ATOH1*, *SPDEF*, *ERN2*, and spliced *XBP1* in primary human colonoids treated with DAPT and 4μ8C. Symbols represent individual biological replicates from 3 independent experiments. Data are represented as mean ± SEM. Mean values were compared by 2-way ANOVA. *P < 0.05; **P < 0.01; ***P < 0.001; ****P < 0.0001.

To determine whether our findings were relevant to humans, we first analyzed gene expression for individuals with ulcerative colitis (UC), as molecular and histologic features of UC resemble the goblet cell, mucus layer, and microbial defects seen in *Ern2^–/–^* mice (34–38). *ERN2* mRNA expression was significantly reduced in rectal biopsies from individuals with UC compared with those without (Figure 8, A and B; ref. 39), and this was highly correlated with reduced expression of *ATOH1* and *SPDEF*. These data implicate loss of ERN2 function as a causal factor in goblet cell depletion associated with UC. To test experimentally whether ERN2 function is involved in human goblet cell development, we assayed DAPT-induced differentiation in primary human colonoids. Treatment with DAPT induced colonoid differentiation, as assessed by loss of spheroid morphology (i.e., flat, unpolarized cells, lack of pronounced actin brush border) and accumulation of a differentiated morphology (i.e., enhanced cell height with basal orientation of nuclei and enhanced apical actin brush border staining) (Figure 8C). Differentiation was accompanied by increased expression of *ATOH1*, *SPDEF*, *ERN2*, and spliced *XBP1* mRNA (Figure 8D). Treatment with 4μ8C to block *XBP1* splicing resulted in a significant reduction in DAPT-induced differentiation and goblet cell gene expression (Figure 8, C and D). From these data, we conclude that ERN2 and XBP1 are important for human goblet cell development and function.

## Discussion

**Figure 9:**
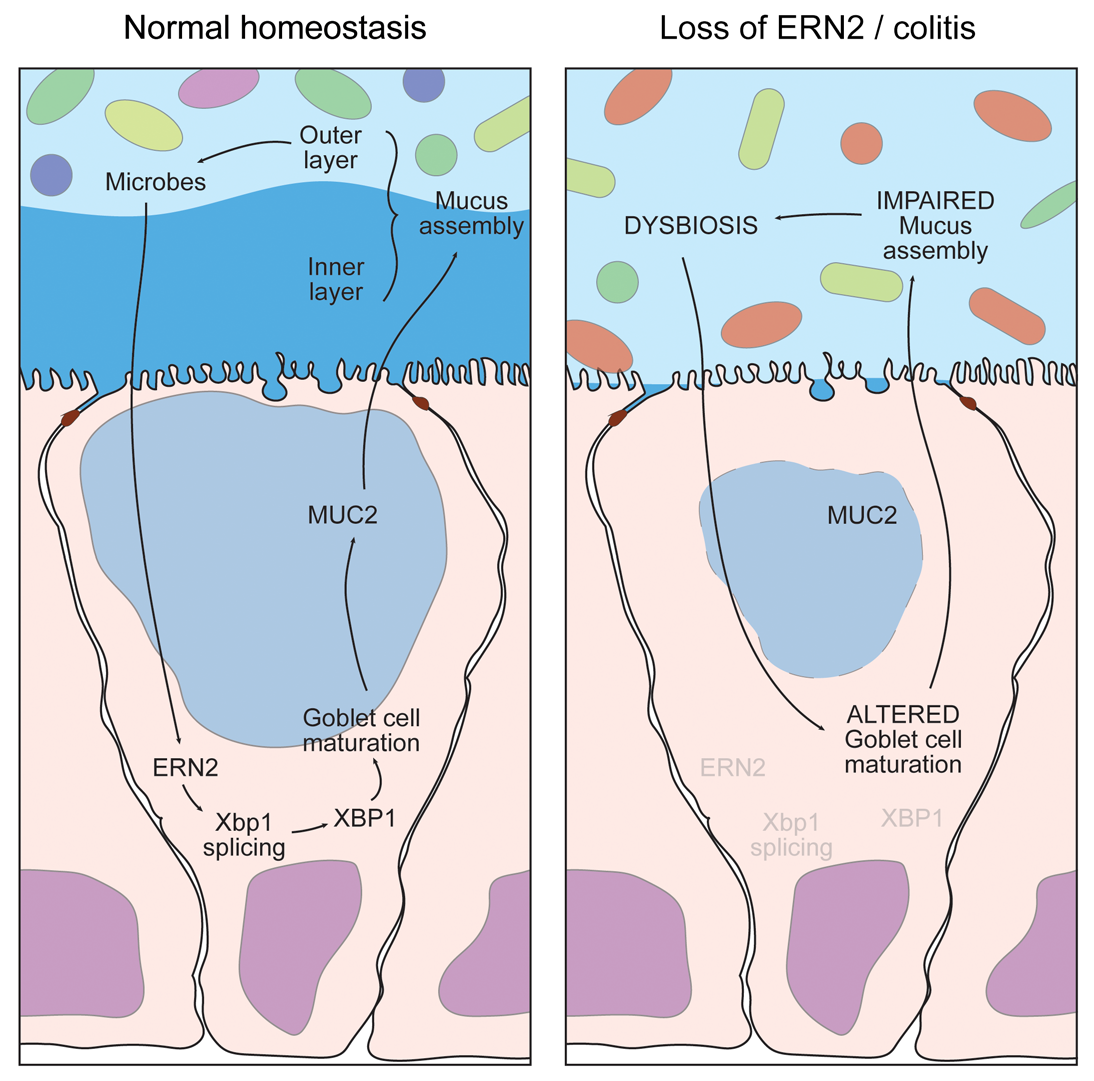
Model for function of ERN2 in microbe-epithelial-mucus feedback loop regulating mucosal homeostasis.

Our results define an essential role for the epithelial-specific ER stress sensor ERN2/IRE1β in the crosstalk between gut microbiota and the colonic epithelium that drives goblet cell differentiation and development of an effective mucus barrier (Figure 9). ERN2 expression and function are indispensable to this process and are induced by gut microbial colonization. In turn, the mucus barrier produced following ERN2 expression selects for a colonizing microbial community that maintains *Ern2* expression and thus goblet cell differentiation and mucus barrier assembly. The ubiquitously expressed ERN1/IRE1α paralogue does not function redundantly to ERN2 in this context. In mice lacking *Ern2* but expressing *Ern1*, goblet cells fail to differentiate in response to microbes, and the defective mucus barrier produced selects for a dysbiotic microbial community that is unable to induce *Ern2* gene expression when transferred into WT recipients. Thus, unlike ERN1, the epithelial-specific ERN2 paralogue enables a crosstalk between host and microbe that is essential for development and maintenance of mucosal tissues.

ERN2 appears to act by splicing *Xbp1* to affect goblet cell maturation and the mucus barrier. This is in line with a broader view of XBP1 function in development of multiple secretory lineages along the GI tract (5, 40, 41), including, as shown here, goblet cells of the colon epithelium. Ribeiro and coworkers also proposed that ERN2 acts via XBP1 in mediating allergen-induced development of mucus-producing cells in the airway epithelium (15), although a more recent study did not identify an ERN2-XBP1 dependence in similar models (42). We cannot, however, rule out the contribution of ERN2 endonuclease activity via RIDD. Kohno and coworkers reported that ERN2 RIDD activity degrades *Muc2* mRNA to posttranscriptionally regulate MUC2 protein expression (and thus ER stress) (6). We did not see accumulation of *Muc2* mRNA in colon crypt epithelial cells at steady state in CONV-*Ern2^–/–^* mice or following colonization of GF-*Ern2^–/–^* mice — as one might expect in colon tissues lacking ERN2-mediated RIDD activity. Rather, crypts from *Ern2^–/–^* mice tended to have decreased levels of *Muc2* mRNA compared with their WT counterparts. But this result is still not conclusive, as we did not assay *Muc2* mRNA stability in our studies, and the decrease in *Muc2* mRNA levels we observed in crypts from *Ern2^–/–^* mice may simply reflect lower goblet cell numbers. ERN2 function via RIDD remains a plausible and additional mechanism of action.

As the production of XBP1 appears to mediate ERN2 effects in the colon, it remains puzzling why ERN1 is unable to fulfill this function in cells lacking ERN2. Two hypotheses come to mind. First, it is possible that ERN2 expressed at high levels acts constitutively to produce low amounts of XBP1 in the absence of ER stress — this expands ER function to maintain homeostasis and prevent ER stress. ERN1 cannot rescue the phenotype because it is expressed at comparatively low levels and requires activation by ER stress to initiate splicing of *Xbp1* mRNA. Though plausible, one flaw in this explanation is that cells lacking ERN2 show evidence of ER stress — so why is ERN1 unable to rescue the phenotype? Perhaps ERN1 cannot sense the level or nature of ER stress induced in goblet cells lacking ERN2. A second possibility could be that ERN2 senses signals in the ER lumen unique to the mucosal surface, signals that remain invisible to ERN1. Though ERN2 fails to respond robustly to disruption of proteostasis by thapsigargin or tunicamycin (16), ERN2 has maintained throughout evolution an ER lumenal domain that may respond to other factors.

Similarly, different hypotheses have been proposed to explain why epithelial cells at mucosal surfaces uniquely express the 2 IRE1 paralogues. As we have noted before, the 2 IRE1 paralogues are found only in vertebrates where the *Ern2* gene likely arose after a whole genome duplication event that typifies vertebrate genomes (43). In one mechanism to retain the duplicated gene, a relaxed selective pressure on *Ern2* may have allowed for the accumulation of sequence variations that enabled the selection of new functions — a process called neofunctionalization. The *Ern2* genes in mammals have acquired the most pronounced sequence variation compared with their *Ern1* counterparts (43). This, we suggest, is a strong hint toward explaining the unique tissue distribution of *Ern2* gene expression. A defining feature of epithelial barriers in mammals, compared with lower vertebrates (where *Ern1* and *Ern2* genes are more similar) and compared with invertebrates (which have only a single IRE1 gene), is the evolution of a mucus-based rather than a chitin-based system of host-environment barrier immunity (43, 44). And this, as we show here, requires ERN2. Though other hypotheses have been proposed to explain why epithelial cells at mucosal surfaces may have evolved to express ERN2 (1, 5, 16, 45, 46), we suggest the evolutionary shift to a mucus-based system of mucosal immunity in mammals underlies its evolution and at least one of its major functions.

Another part of the neofunctionalization of *Ern2* genes could have been driven by the need to quench ERN1 hyperactivation in the context of many, diverse, and continuous exposures to cell stress inherent to mucosal surfaces (1, 16). We previously found that the endonuclease activity of ERN1 is more potent, and unlike ERN2, it requires activation by ER stress (16). Such a chronic need for stress-induced ERN1 activation to fulfill goblet cell development in the intestine and other tissues forming mucosal barriers would likely be accompanied by chronic inflammation (5, 41). And in addition, upon induction of cell stress, we have found that ERN2 assembles with ERN1 to inhibit its activation. This could also serve as a selective factor driving the evolution of 2 IRE1 paralogues that diverged in mammals.

How gut microbes regulate *Ern2* gene expression remains unknown. Our results suggest that specific components of the microbiota are needed to turn on goblet cell development and ERN2 function. Butyrate appears to be one component that can activate this process, although not necessarily the only component. How loss of ERN2 function enables selection of a microbial community that fails to induce *Ern2* expression and function also remains unknown. A likely explanation is that *Ern2^–/–^* mice produce altered mucins and defective mucus barriers that select for microbial species that thrive independently of ERN2 and its downstream effector functions. Our studies show, for example, that crypts from *Ern2^–/–^* mice have reduced expression of sulfo- and sialyltransferase genes involved in mucin glycosylation. The composition of their mucus barriers likely mimics the mucin glycans produced by GF mice (e.g., shorter, less complex glycans) (47). Microbes that favor such a mucin environment would not benefit from induction of *Ern2* expression or goblet cell maturation and mucus assembly — thus explaining the dysbiosis and its phenotype with respect to *Ern2* expression.

Finally, we note that the colon epithelial phenotype observed in *Ern2^–/–^* mice contains features mimicking the human disease UC, including defects in goblet cell maturation and mucin secretion, impaired assembly of the colon mucus layer, and dysbiosis of the gut microbiota (34–38). While the epithelium still develops as an intact monolayer in *Ern2^–/–^* mice, the altered structure and function of the mucosal surface is associated with earlier onsets of gut infection, chemical injury, and inflammation. Thus, the *Ern2^–/–^* mice, and in particular the microbiota-epithelial-mucus barrier feedback loop enabled by ERN2, may model epithelial defects associated with the onset of inflammation in UC. Consistent with this idea, we found *ERN2* mRNA expression was reduced in individuals with UC (39), and stimulating ERN2 enzymatic activity using small molecule activators (48–51) or promoting *ERN2* expression by administering consortia of microbes or their products may enable the restoration of mucus barrier function and normalize host-microbiota crosstalk in some disease contexts.

## Methods

### In vivo experiments in mice

#### Husbandry and breeding.

C57BL/6J WT mice (000664, The Jackson Laboratory), *Ern2^–/–^* mice (a gift from M. Hussain [State University of New York Downstate Medical Center, Brooklyn, New York, USA] with permission from D. Ron [University of Cambridge, Cambridge, United Kingdom]), *Xbp1^fl/fl^* mice (a gift from L. Glimcher [Dana-Farber Cancer Institute, Boston, Massachusetts, USA]) and Tg(*Vil1*-cre)1000Gum mice (021504, The Jackson Laboratory) were maintained in a specific pathogen–free facility under standard conditions. WT and *Ern2^–/–^* mice were rederived GF and maintained in sterile isolators with autoclaved bedding, food, and water. Littermate comparisons were performed between siblings derived from *Ern2^–/–^* and WT matings. Cohousing comparisons were performed by weaning age–matched WT weanlings with *Ern2^–/–^* weanlings from independent homozygous matings into the same cage at 3 weeks of age. Mice with intestine-specific deletion of *Xbp1* were generated by first mating *Xbp1^fl/fl^* mice with *Vil-Cre*^+^ mice followed by crossing of *Xbp1^fl/+^*;*Vil1-Cre*^+^ mice with *Xbp1^fl/fl^* mice to generate *Xbp1^fl/fl^*;*Vil1-Cre*^+^ and *Xbp1^fl/fl^*;*Vil1-Cre*^–^ mice for experiments.

#### DBZ studies.

Cohoused 8-week-old WT and *Ern2^–/–^* mice were interperitoneally injected daily for 4 days with 10 μmol/kg DBZ (Chiralix, previously Syncom) suspended in 0.5% (w/v) Methocel E4M (Dupont) and 0.1% Tween 80 in water (17). Control mice were injected daily with vehicle alone. On day 5, mice were sacrificed, a segment of distal colon was collected for histologic analysis, and the remaining distal colon was used for epithelial cell collection.

#### C. rodentium studies.

Cohoused 6- to 8-week-old WT and *Ern2^–/–^* mice were fasted for 4 hours and then gavaged with approximately 2 to 4 × 10^8^ CFUs *C*. *rodentium* (strain DBS100) from an overnight culture. All procedures with mice were performed in a biosafety cabinet, and mice were housed in a biosafety level 2 room for the duration of the experiments. For quantification of *C*. *rodentium* in stool, fresh stool pellets were homogenized with a sterile pestle in 0.5 mL PBS, plated on MacConkey agar plates, and incubated overnight at 37°C. For quantification of *C*. *rodentium* from colon tissue, fresh or snap-frozen colon tissue segments were homogenized with a sterile pestle in PBS, plated on MacConkey agar plates, and incubated overnight at 37°C. *C*. *rodentium* CFUs were determined by counting colonies from triplicate plating of a serial dilution and normalizing the number of colonies by stool or tissue weight. At defined time points during the infection, mice were sacrificed, colon tissue was collected to determine *C*. *rodentium* load, and a segment of distal colon was collected for histologic analysis. H&E-stained sections were evaluated by a blinded pathologist for mucosal hyperplasia, goblet cell depletion, crypt apoptosis, epithelial erosion, crypt architectural distortion, lymphocytic infiltrate intensity, neutrophilic infiltrate intensity, and submucosal inflammation and edema. Each category was assigned a score of 0 to 3 and summed to give a total histology score for each individual animal.

#### DSS-associated colitis studies.

Separately housed 6- to 8-week-old WT and *Ern2^–/–^* mice were given DSS (2%, Alfa Aesar, catalog J63606-3S, lot U03C023) in sterile drinking water for 8 days. After 8 days, mice were placed back on regular sterile drinking water for a recovery phase. Control mice received sterile drinking water throughout. Mice were weighed daily and evaluated for fur quality, posture, activity, stool consistency, and bleeding. Mice with more than 20% weight loss were removed from the study and euthanized.

#### Colonization of GF mice with a gut microbiota.

GF mice were colonized with gut microbes derived from CONV-WT or CONV-*Ern2^–/–^* donor mice. Donor microbiotas were prepared by harvesting the cecum from donor mice, cutting open longitudinally, scraping cecal contents with forceps into a tissue grinder tube, and homogenizing in 2.5 mL sterile PBS. The cecal homogenate was centrifuged at 500*g* for 3 minutes, and the supernatant was immediately used for colonization into recipient mice. Recipient mice were colonized by 1-time gavage with 200 μL of cecal homogenate and housed for the duration of the experiment in sterile cages outside of the GF isolator. At 14 days after colonization, mice were sacrificed, a segment of distal colon was collected for histologic analysis, and the remaining distal colon was used to isolate colon crypt epithelial cells.

#### Colonization of GF mice followed by TUDCA administration.

WT and *Ern2^–/–^* GF mice were colonized with a microbiota from WT donor mice as described above. Beginning the day after colonization, mice were gavaged every other day for 12 days with 500 mg/kg TUDCA in PBS (Sigma-Aldrich) (23). At 14 days after colonization, mice were sacrificed, and a segment of distal colon was collected for histologic analysis.

#### Antibiotic treatment followed by cohousing to recolonize gut microbes.

WT mice were treated with an antibiotic cocktail (0.5 g/L ampicillin, 0.25 g/L vancomycin, 0.5 g/L metronidazole, and 0.25 g/L neomycin [Sigma-Aldrich] dissolved in drinking water) for 7 days (52). After 7 days, antibiotic-treated mice were recolonized by cohousing with CONV-WT or *Ern2^–/–^* donor mice for 14 days. At 14 days after colonization, mice were sacrificed, and a segment of distal colon was collected for histologic analysis. Colon tissue was also collected from age-matched mice that did not receive antibiotics and mice that received antibiotics for 7 days, but without subsequent cohousing. For studies involving DSS after cohousing, WT and *Ern2^–/–^* mice were treated with antibiotics, as described above, followed by cohousing with WT or *Ern2^–/–^* donors for 14 days. After 14 days, mice were treated with DSS (2% in drinking water) for 8 days, followed by recovery (regular water) for 14 days. Mice were weighed daily and evaluated for clinical symptoms of fur quality, posture, activity, stool consistency, and bleeding. WT and *Ern2^–/–^* mice that did not receive antibiotics or cohousing were treated with DSS as controls.

#### Sodium butyrate administration in GF mice.

WT and *Ern2^–/–^* GF mice received either regular drinking water or drinking water supplemented with 100 mM sodium butyrate (A11079 Alfa Aesar) (33) for 14 days. Mice were monitored for normal drinking behavior. After 14 days, mice were sacrificed, and a segment of distal colon was collected for histologic analysis.

#### Isolation of colon epithelial cells and intact colon crypts.

Colon tissue was harvested, lumenal contents were gently removed, and the tissue was flushed with ice-cold PBS. The tissue was cut open longitudinally, cut into small pieces, washed 3 times with ice-cold PBS, and incubated in PBS with 10 mM EDTA for 45 minutes at 4°C on a rotary shaker. Epithelial cells were dissociated by vigorous shaking for 5 minutes, and the cell suspension was decanted into base media (Advanced DMEM/F12 Media [Gibco, Thermo Fisher Scientific], 20% FBS, 10 mM HEPES, 100 μg/mL penicillin/streptomycin, 2 mM l-glutamax). The remaining tissue pieces were subjected to a second round of epithelial cell isolation in the same manner. To isolate intact colon crypts, the epithelial cell suspension was passed through a 100 μm strainer followed by a 40 μm strainer. Intact crypts retained on the 40 μm strainer were washed, eluted with base media, and pelleted by centrifugation at 300*g* for 3 minutes. Crypts were either used to generate organoid lines as described below or washed with PBS and stored at –80°C for later use.

### In vitro experiments with colonoids and cell lines

#### DAPT-induced differentiation in mouse colonoid lines.

Three independent epithelial colonoid lines were prepared from WT and *Ern2^–/–^* mice. Intact colon crypts were harvested from mice as described above. Crypts were resuspended in Matrigel (Corning) on ice, and 30 μL drops were plated in 24-well plates (typically 3 to 4 drops for the initial isolation) and cultured in complete media (base media plus 50% WRN-conditioned media prepared from L cells expressing Wnt/R-spondin/Noggin as described, ref. 53) with 10 μM Y27632 (ROCK inhibitor, Sigma-Aldrich, Y0503) at 37°C 5% CO_2_. Media was changed every other day. Cultures were passaged by dissolving Matrigel with Cell Recovery Solution (Corning), washing colonoids with base media, mechanically disrupting colonoids by pipetting, and plating in 1.5- to 2-fold more Matrigel than used in the previous plating. Cultures were expanded as needed for experiments and cryopreservation of colonoid lines. For differentiation experiments, WT and *Ern2^–/–^* colonoids were plated in Matrigel and cultured in complete media supplemented with 10 μM Y27632 for 24 hours. After 24 hours, colonoids were treated with 10 μM DAPT (Sigma-Aldrich, 565770) ± 50 μM 4μ8C (Sigma-Aldrich, 412512) for an additional 24 hours; control colonoids had complete media alone or complete media with 50 μM 4μ8C (no DAPT). Bright-field images of colonoids were collected at 3 different focal planes over the Matrigel drop using a Cytation 5 imager (BioTek). Colonoids that were in focus were scored as either spheroid or nonspheroid, the percentage of colonoids with a spheroid morphology was calculated for each condition, and fractional change in the percentage of spheroid morphology was expressed relative to the control colonoids (no DAPT, no 4μ8C). The experiment was performed on 2 to 3 independent colonoid lines with at least 2 independent experiments for each line. Colonoids were collected for RNA extraction and expression analysis of goblet cell marker genes by quantitative PCR (qPCR).

#### DAPT-induced differentiation in human colonoid line.

A human colonoid line (H514) was obtained from the Harvard Digestive Disease Center Organoid Core. Colonoids were cultured as described above for mouse colonoids, except they were maintained in human colon complete media (advanced DMEM/F12 supplemented with 60% WRN conditioned media, 1× GlutaMAX, 10 mM Hepes, 100 μg/mL primocin [Invivogen, ant-pm-2], 100 μg/mL normocin [Invivogen, ant-nr-2], 0.5X B27 [Gibco, Thermo Fisher Scientific, 12587010], 0.5X N2 [Gibco, Thermo Fisher Scientific, 17502-048], 10 mM nicotinamide [MilliporeSigma, N0636], 0.5 mM *N*-acetyl-cysteine [MilliporeSigma, A8199], 0.5 μM A83-01 [MilliporeSigma, SML0788], 3.3 μg/mL SB202190 [MilliporeSigma, S7067], 50 ng/mL EGF [Peprotech, 315-09], 10 nM gastrin [MilliporeSigma, G9145], and 100 nM prostaglandin E2 [MilliporeSigma, Y0503]). For differentiation experiments, human colonoids were cultured in human colon complete media for 72 hours prior to treating with 10 μM DAPT (Sigma-Aldrich, 565770) with or without 50 μM 4μ8C (Sigma-Aldrich, 412512) for an additional 48 hours; control colonoids had human colon complete media alone or with 50 μM 4μ8C (no DAPT). One set of colonoids was used for RNA extraction. A second set of colonoids (plated in black plates with glass bottoms, Cell Vis P24-0-N) was fixed with 4% paraformaldehyde (1 hour at room temperature), washed 1× with 0.3 M glycine (30 minutes), and washed 3× with PBS. Fixed colonoids were permeabilized and blocked in PBS containing 1% Triton X-100 and 5% BSA (1.5 hours at room temperature). Colonoids were washed 3× in IF Buffer (PBS, 0.1% BSA, 0.2% Triton X-100, 0.1% Tween 20) and stained with Phalloidin 647 (Abcam ab176759, 1:100 dilution in IF Buffer, 1 hour) followed by DAPI (2 μg/mL in IF Buffer, 5 minutes). Colonoids were imaged on a Biotek Cytation 5 imager using a 10× objective. Images were collected over the entire well and stitched together for morphological analysis of differentiated colonoids based on cell height, basal localization of nuclei, and brush border actin staining.

#### Butyrate-induced Ern2 expression in epithelial cell lines.

T84 cells were maintained in 1:1 DMEM/F12 media supplemented with 6% newborn calf serum. Cells were plated on 0.33 cm^2^ Transwell inserts with 3 μm pore size polyester membranes and allowed to polarize for 7 days. Monolayer formation was assessed by measuring transepithelial electrical resistance with an epithelial volt/ohm meter (EVOM; World Precision Instruments). Monolayers were treated with 10 mM sodium butyrate, 10 mM sodium acetate, or media alone for 24 hours. LS174T cells were cultured in DMEM supplemented with 10% FBS and 1× nonessential amino acid solution (Gibco, Thermo Fisher Scientific). Cells were treated with 1 mM sodium butyrate or control media for 24 hours. After cell treatments, cells were washed with PBS and RNA was extracted for expression analysis.

#### ERN2 Xbp1 splicing activity in HEK293^doxERN2^ cell line.

The HEK293^doxERN2^ cell line was described previously (16). Expression of Flag-tagged human ERN2 was induced by treatment with 0, 10, or 100 ng/mL doxycycline for 24 hours. RNA was extracted for expression analysis by qPCR (spliced *Xbp1* transcript) and RNA-Seq as described below.

### Histologic analysis of mouse colon tissues

#### Histologic analysis of colon crypt length and goblet cells.

A segment (1 cm) of distal colon was isolated, lumenal content was gently removed, and the tissue was fixed in 10% formalin for 24 to 48 hours at room temperature. Fixed tissue was embedded in paraffin and sections prepared for histologic staining with H&E or AB. Sections were also stained with anti-Ki67 (Cell Signaling Technologies, catalog CST12202S, 1:500 dilution) and anti-MUC2 (Santa Cruz Biotechnology Inc., catalog sc-15334, 1:100 dilution) antibodies and Cy3-labeled anti-rabbit secondary antibodies. Images of stained sections were collected on an Olympus BX41 microscope with a Pixelink PL-D682CU color CMOS camera for brightfield and epifluorescence imaging. Crypt length was measured in H&E- or AB-stained sections in well-oriented crypts that extended the full length of the mucosa. Goblet cells were enumerated by counting AB^+^, PAS^+^, or anti-MUC2^+^ mucin granules in well-defined crypts and normalizing by the total number of IECs (stained nuclei) lining the crypt. In most cases, crypt length and goblet cell numbers were averaged over at least 5 well-defined crypts. Goblet cell theca area was measured in ImageJ (NIH) by measuring the AB-stained area for goblet cells in the upper half of well-oriented crypts.

#### Histologic analysis of colon mucus layer.

To preserve the colon mucus layer for analysis, a 2 to 3 cm segment of distal colon containing a fecal pellet was excised and fixed in Carnoy’s solution (60% methanol, 30% chloroform, 10% acetic acid) for 24 to 48 hours at room temperature (54). Fixed tissue was washed twice with 100% methanol, followed by 2 washes with 100% ethanol. The fixed tissue segment was cut into pieces and embedded in paraffin for cross-section cuts through the fecal pellet. No water was used in any of the fixation or processing steps to ensure that the mucus layer was preserved. Slides were stained with AB/PAS. The inner mucus layer was analyzed by measuring thickness of the AB/PAS-stained region adjacent to the epithelium at regularly spaced intervals in images covering the entire section around a fecal pellet (usually in 2 to 3 slices of a single fecal pellet) and averaged over the entire distribution of measurements for an individual animal. Carnoy’s fixed tissue was also stained with the in situ hybridization probe EUB338 to detect lumenal bacteria as described (54). Slides were deparaffinized, stained with 20 mg/mL of 594-EUB338 probe (Alexa Fluor 594–5′-GCTGCCTCCCGTAGGAGT-3′, Integrated DNA Technologies) or 594-CONTROL probe (Alexa Fluor 594–5′-CGACGGAGGGCATCCTCA-3′, Integrated DNA Technologies) in hybridization buffer (20 mM Tris pH 7.4, 0.9 M NaCl, 0.1% [w/v] SDS) at 50°C overnight in a humidified chamber. After staining, slides were incubated in FISH washing buffer (20 mM Tris pH 7.4, 0.9 M NaCl) for 20 minutes at 50°C, then washed 3 times with PBS. Sections were costained with DAPI (D3571, Thermo Fisher), and slides were mounted using Prolong Antifade (P36961, Thermo Fisher).

### Molecular analysis of mRNA gene expression

#### Expression analysis by RNA-Seq.

RNA was extracted from intact colon crypts and cell lines using the RNeasy Mini Extraction Kit (QIAGEN). For RNA-Seq, library preparation, next-generation sequencing, and data processing were performed by the Dana-Farber Cancer Institute Molecular Biology Core Facility. Total RNA quality and concentration were measured using Aligent Bioanalyzer, and RNA libraries were prepared using KAPA stranded mRNA HyperPrep Kits. RNA was sequenced by synthesis with 75 cycles of single end reads on an Illumina NextSeq500. Data were processed using the Visualization Pipeline for RNA-Seq (VIPER) (55). Sequence reads were aligned using STAR (56), and raw gene counts were used to calculate differential expression (log_2_ [group1/group2]) between groups with DESeq2 (57). Genes with genome-wide *P_adj_* < 0.01 were considered significantly different. Gene set enrichment analysis was performed by calculating hypergeometric distributions for differentially expressed genes (*P_adj_* < 0.01) using epithelial cell signatures derived from Haber et al. (2). Functional analysis of differentially expressed genes was performed using gProfiler (58). RNA-Seq data sets were deposited in the NCBI’s Gene Expression Omnibus (GEO GSE205701 and GSE205721).

#### Expression analysis by qPCR.

RNA was extracted from intact colon crypts, colon epithelial cells, colonoids, and cell lines using the RNeasy Mini Extraction Kit (QIAGEN). cDNA was prepared from total RNA using the iScript cDNA Synthesis Kit (Bio-Rad). Target gene cDNA was amplified using primers (Supplemental Table 10) and Sso Advanced Universal SYBR Green Supermix (Bio-Rad) on a CFX384 Touch Real-Time PCR Detection system (Bio-Rad). Reactions were assayed in triplicate for each sample, and the average Cq value was used to calculate the mean expression ratio of the test sample compared with the control sample using the 2-ΔΔCt method. Cq values for targets were analyzed relative to Cq values for *Hprt* and *Ppia* reference genes.

### Molecular and biochemical analysis of mouse gut microbiome

#### Metagenomic sequencing.

Stool pellets from WT and *Ern2^–/–^* mice were collected into sterile tubes, frozen on dry ice, and stored at –80 °C. DNA was extracted using ZymoBIOMICS DNA Miniprep Kit (D4300, Zymo Research) and metagenomic DNA-Seq libraries were constructed using the Nextera XT DNA Library Prep Kit (FC-131-1096, Illumina) and sequenced on a NextSeq500 Sequencing System as 2 × 150 nucleotide paired-end reads. Shotgun metagenomic reads were first trimmed and quality filtered to remove sequencing adapters and host contamination using Trimmomatic (59) and Bowtie2 (60), respectively, as part of the KneadData pipeline (https://huttenhower.sph.harvard.edu/kneaddata/). Metagenomic data were profiled for microbial taxonomic abundances and microbial metabolic pathways using Metaphlan3 (61) and HUMAnN3 (62), respectively. Sequencing data were deposited in the Sequence Read Archive (SRA PRJNA846320).

#### Short chain fatty acid analysis.

Stool pellets from WT and *Ern2^–/–^* mice were collected directly into sterile preweighed tubes, immediately frozen, and stored at –80°C. Pellets were homogenized in HPLC grade water by vortexing. The pH of the cleared homogenate was adjusted to 2-3, 2-methyl pentanoic acid was added as an internal standard (0.1%), and short chain fatty acids (SCFAs) were extracted by adding 1 volume of ethyl ether anhydrous. Samples were vortexed for 2 minutes and centrifuged at 5000*g* for 2 minutes. The upper ether layer was collected, and SCFA content was analyzed on an Agilent 7890B gas chromatography system with flame ionization detector using a high-resolution capillary column for detection of volatile acids (DB-FFAP, 30 m × 0.25 mm with 0.25 μm film thickness; Agilent Technologies). A standard solution containing 10 mM of acetic, propionic, isobutyric, butyric, isovaleric, valeric, isocaproic, caproic, and heptanoic acids (Supelco CRM46975) was processed and analyzed in the same manner as the stool samples. The retention times and peak heights of the acids in the standard mix were used as references for the unknown samples. Each acid was identified by its specific retention time, and the concentration was determined and expressed as mM per gram of fecal material. Chromatograms and data integration were carried out using OpenLab ChemStation Software (Agilent Technologies).

### Statistics

Unless otherwise indicated in figure legends, figures include all independent measures shown as symbols, and bars represent mean values ± SEM. Mean values between 2 groups were compared using unpaired Student’s *t* test (2 sided), with multiple comparisons corrected using the FDR approach of Benjamini, Krieger, and Yekutieli. In all cases, data distribution was assumed to be normal, but with no assumption about consistent SDs. For comparisons of 3 or more groups, mean values were compared using 1-way or 2-way ANOVA as appropriate, with multiple comparisons corrected using statistical hypothesis testing (Tukey’s). Survival curves were compared using a log rank test. All analyses were performed in Prism (GraphPad Software).

### Study approval

All experimental procedures involving mice were approved by Boston Children’s Hospital Institutional Animal Care and Use Committee.

## Author contributions

MJG and WIL conceived the project. MJG, HDL, DVW, KBG, SEF, JR Thiagarajah, BAM, JR Turner, and WIL contributed to the experimental design. MJG, HDL, DVW, and IAMK conducted experiments and processed data. MJG, HDL, JR Turner, and WIL analyzed data and interpreted results. MJG, HDL, and WIL wrote the manuscript. All authors reviewed the manuscript prior to submission.

## Supplementary Material

Supplemental data

Supplemental data sets 1-8

Supplemental tables 1-10

## Figures and Tables

**Table 1:**
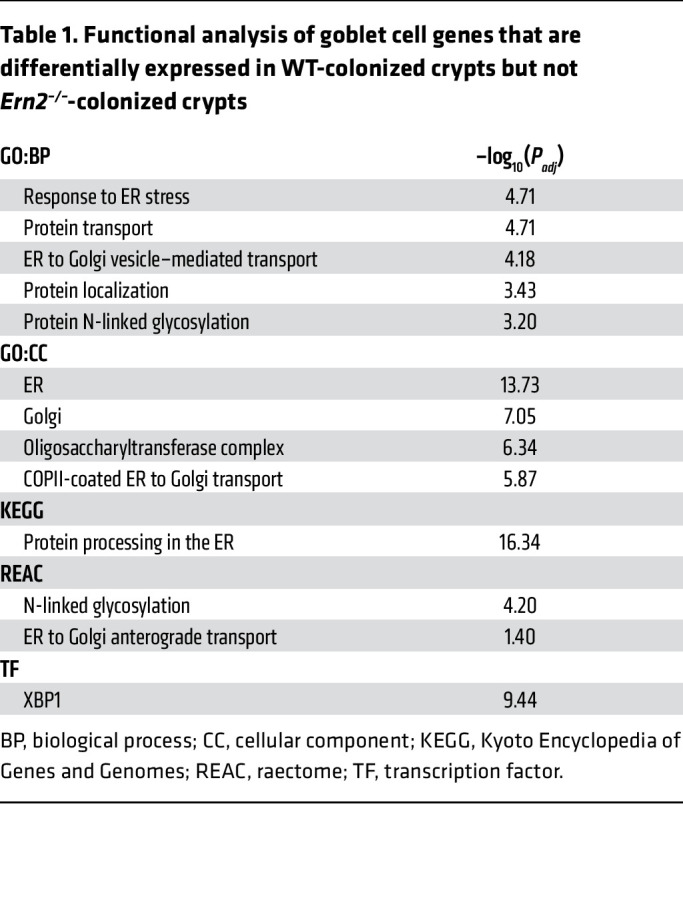
Functional analysis of goblet cell genes that are differentially expressed in WT-colonized crypts but not Ern2^–/–^-colonized crypts

## Version 1 06/21/2022

In-Press Preview

## Version 2 09/01/2022

Electronic publication
