# Supplementary material for: The epithelial-specific ER stress sensor ERN2/IRE1β enables host-microbiota crosstalk to affect colon goblet cell development
Source: J Clin Invest. 2022 Sep 1;132(17):e153519. doi: 10.1172/JCI153519 (PMC9435652; doi:10.1172/JCI153519)
Supplement: Supplemental data [file jci-132-153519-s086.pdf]

The epithelial-specific ER stress sensor ERN2/IRE1β enables host-microbiota crosstalk to affect colon goblet cell development

Michael J. Grey[1,2,3,9], Heidi De Luca[1], Doyle V. Ward[4,5], Irini A. M. Kreulen[1,8], Katlynn Bugda Gwilt[1], Sage E. Foley[4,5], Jay R. Thiagarajah[1,2,3], Beth A. McCormick[4,5], Jerrold R. Turner[3,6,7], and Wayne I. Lencer[1,2,3,10]

[1]Division of Gastroenterology and Nutrition, Boston Children's Hospital, Boston, Massachusetts, USA

[2]Department of Pediatrics, Harvard Medical School, Boston, Massachusetts, USA

[3]Harvard Digestive Disease Center, Boston Children's Hospital, Boston, Massachusetts, USA

[4]Department of Microbiology and Physiological Systems, University of Massachusetts Chan Medical School, Worcester, Massachusetts, USA

[5]Program in Microbiome Dynamics, University of Massachusetts Chan Medical School, Worcester, Massachusetts, USA

[6]Laboratory of Mucosal Barrier Pathobiology, Department of Pathology, Brigham and Women's Hospital, Boston, Massachusetts, USA

[7]Departments of Pathology and Medicine, Harvard Medical School, Boston, Massachusetts, USA

[8]Present address: Tytgat Institute for Liver and Intestinal Research, Department of Gastroenterology and Hepatology, Amsterdam University Medical Centers, University of Amsterdam, Amsterdam, The Netherlands

[9]Co-corresponding author: Michael J. Grey, 300 Longwood Avenue, Enders 606, Boston, MA 02115, 617-583-0463, michael.grey@childrens.harvard.edu

[10]Co-corresponding author: Wayne I. Lencer, 300 Longwood Avenue, Enders 609.2, Boston, MA 02115, 617-919-2573, wayne.lencer@childrens.harvard.edu

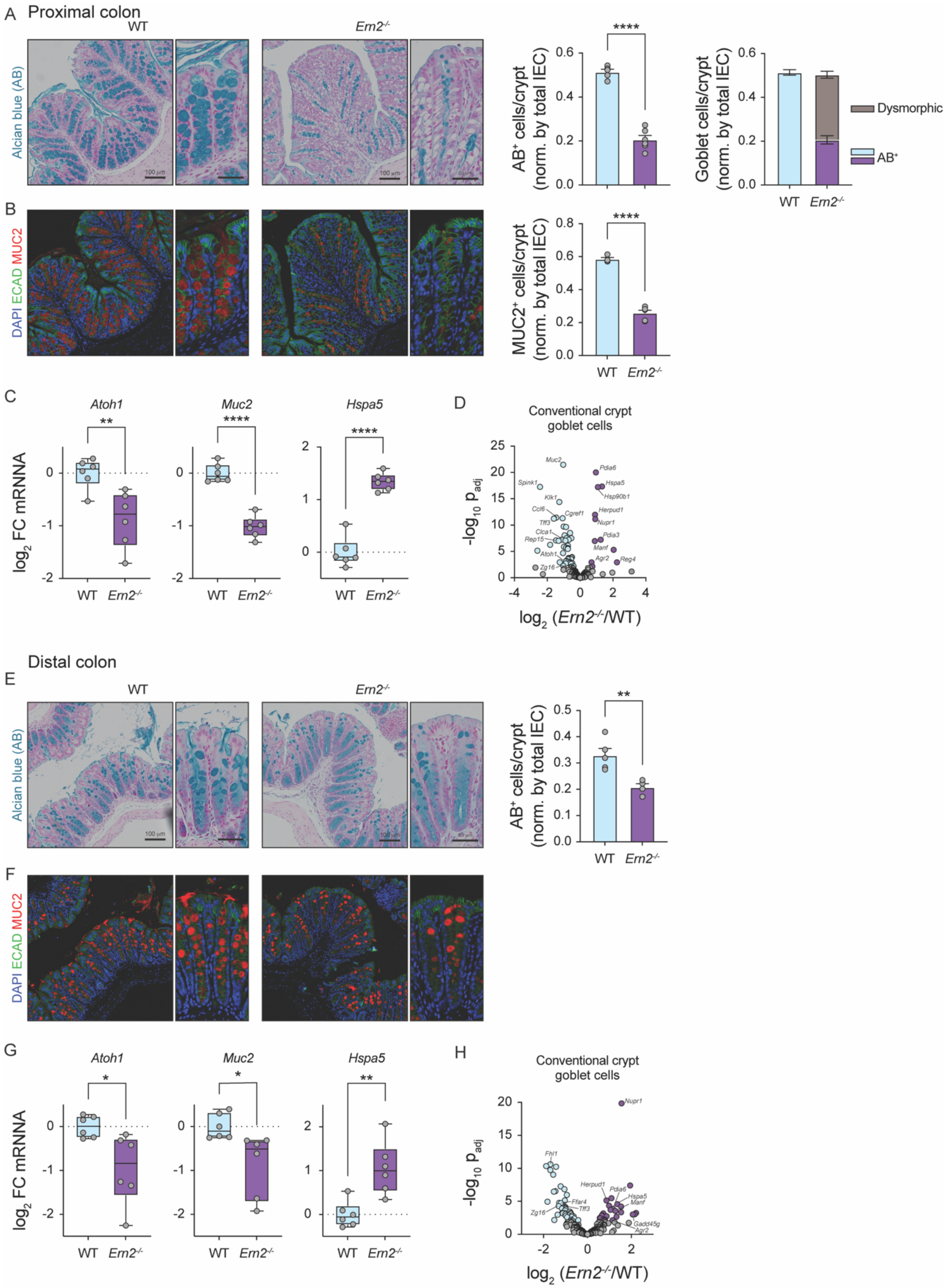
A Proximal colon
WT
Ern2-/-
Alcian blue (AB)
AB+ cells/crypt (norm. by total IEC)
Goblet cells/crypt (norm. by total IEC)
Dysmorphic
AB+
****
B
DAPI ECAD MUC2
MUC2+ cells/crypt (norm. by total IEC)
C
Atoh1
Muc2
Hspa5
log2 FC mRNA
D
Conventional crypt goblet cells
-log10 padj
log2 (Ern2-/-/WT)
Distal colon
E
F
G
H

Supplemental Figure 1 (supporting Figure 1). Histologic and molecular analysis of (A-D) proximal and (E-H) distal colon of CONV-WT and CONV-*Ern2*$^{-/-}$ mice. Representative images of (A,E) Alcian blue (AB) and (B,F) anti-MUC2 antibody-stained sections from CONV-WT and CONV-*Ern2*$^{-/-}$ mice (separately housed). Bar graphs show the number of AB^{+} and $MUC2^{+}$ cells per crypt (total count in entire crypt normalized by number of crypt epithelial cells). Symbols represent the average values for individual mice. Bars represent mean ± SEM. Mean values were compared by unpaired t-test. (C,G) Box plots show relative mRNA expression levels of goblet cell marker genes measured by RNA-seq in colon epithelial cells. Symbols represent individual mice, boxes represent quartiles, and bars represent median and range. (D,H) Volcano plot shows differential expression for conventional crypt goblet cell genes. Significant values ($p_{adj} < 0.01$) are colored purple (increased in *Ern2*$^{-/-}$) or light blue (decreased in *Ern2*$^{-/-}$).

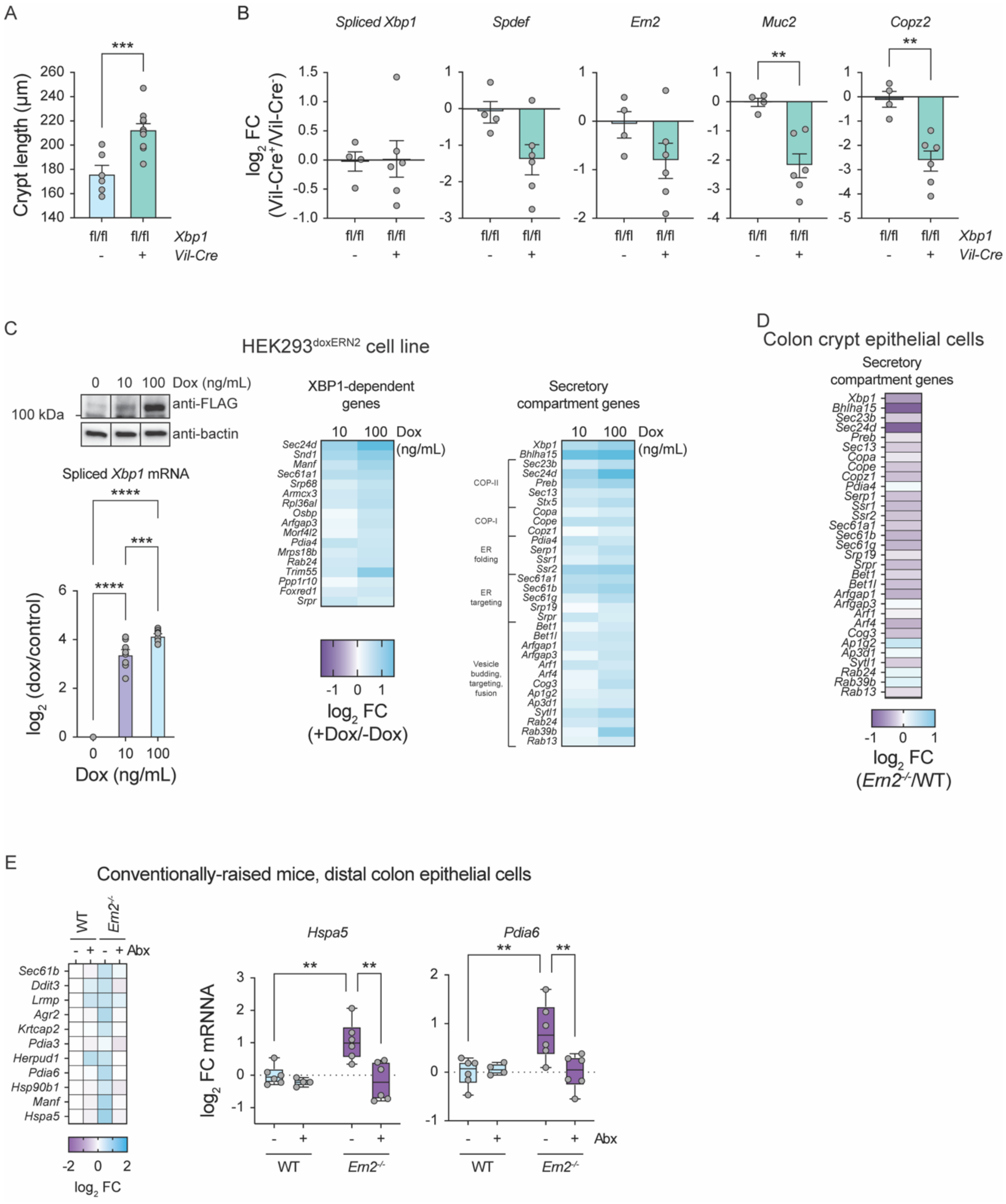

Supplemental Figure 2 (supporting Figure 3) (A,B) Bar graphs shows (A) crypt length measured in well-oriented crypts and (B) relative mRNA expression levels of goblet cell marker genes measured by qPCR in colon crypt epithelial cells isolated from CONV-*Xbp1*$^{fl/fl}$;*Vil-Cre*$^{-}$ and CONV-*Xbp1*$^{fl/fl}$;*Vil-Cre*$^{+}$ mice. Symbols represent relative individual mice and bars represent mean ± SEM. Mean values were compared by unpaired t-test. (C, top left panel) Representative

western blot showing doxycycline-induced expression of FLAG-tagged ERN2 in HEK293^{doxERN2} cell line. The indicated slices all come from the same gel and membrane that was cropped to exclude intervening lanes that contain other treatment conditions not considered here. (bottom left panel) Bar graph shows the fold change in relative expression of spliced *Xbp1* transcript in HEK293^{doxERN2} cells (1) treated with doxycycline at indicated concentration. Symbols represent independent experiments and bars represent mean ± SEM. Mean values compared by one-way ANOVA (n = 9). Data in left panel are reproduced from Grey et al. (1). Heat maps show mRNA expression (relative to uninduced control cells) for genes associated with XBP1 and genes associated with secretory compartment function (n = 3 per group). (D) Heat map shows relative mRNA expression for secretory compartment genes (from (C)) in colon crypt epithelial cells of CONV-WT and CONV-*Ern2*$^{-/-}$ mice. (E) Heat map shows relative mRNA expression of genes indicative of ER stress (chaperones, ERAD, UPR) in epithelial cells from distal colon of CONV-WT and CONV-*Ern2*$^{-/-}$ mice treated with and without antibiotics (Abx). Expression is shown as log2 fold change relative to control CONV-WT mice (no antibiotics). Box plots show relative mRNA expression levels of indicated genes. Symbols represent relative expression for individual mice, boxes represent quartiles, and bars represent median and range. Differences in expression between groups was compared by two-way ANOVA.

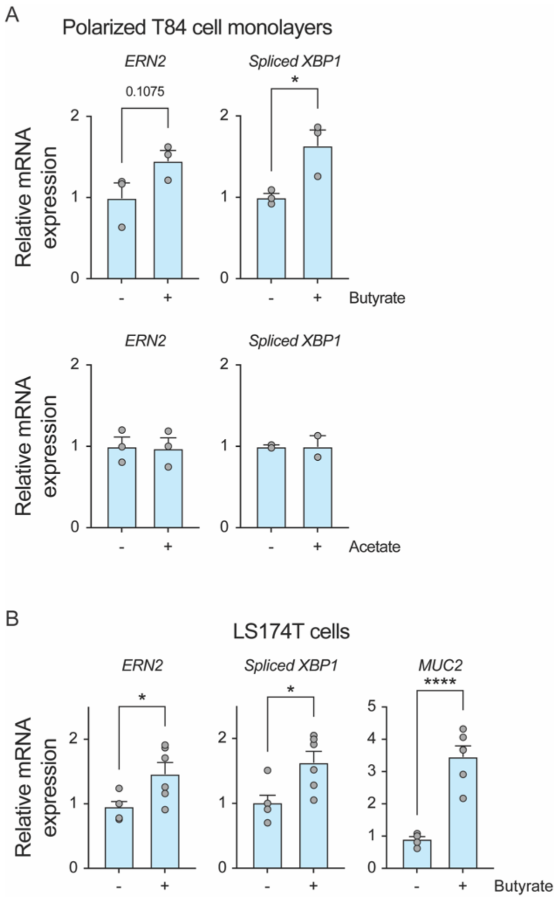

Supplemental Figure 3 (supporting Figure 7). Bar graphs show relative expression of *Ern2* and spliced *Xbp1* mRNA measured by qPCR in (A) polarized T84 cell monolayers (*n* = 3) and (B) LS174T cells (*n* = 6) treated with butyrate or acetate as indicated. Symbols represent independent experiments and bars represent mean ± SEM; mean values were compared by unpaired t-test.

References

1. Grey MJ, Cloots E, Simpson MS, LeDuc N, Serebrenik YV, De Luca H, et al. IRE1β negatively regulates IRE1α signaling in response to endoplasmic reticulum stress. *The Journal of cell biology.* 2020;219(2).
